# Delineation of the pan-proteome of fish-pathogenic *Streptococcus agalactiae* strains using a label-free shotgun approach

**DOI:** 10.1186/s12864-018-5423-1

**Published:** 2019-01-07

**Authors:** Guilherme Campos Tavares, Felipe Luiz Pereira, Gustavo Morais Barony, Cristiana Perdigão Rezende, Wanderson Marques da Silva, Gustavo Henrique Martins Ferreira de Souza, Thiago Verano-Braga, Vasco Ariston de Carvalho Azevedo, Carlos Augusto Gomes Leal, Henrique César Pereira Figueiredo

**Affiliations:** 1 AQUACEN – National Reference Laboratory of Aquatic Animal Diseases, Ministry of Agriculture, Livestock and Food Supply, Veterinary School, Federal University of Minas Gerais, Belo Horizonte, MG Brazil; 2 Laboratory of Cellular and Molecular Genetics, Institute of Biological Sciences, Federal University of Minas Gerais, Belo Horizonte, Brazil; 3 Waters Technologies Brazil, MS Applications Laboratory, Waters Corporation, São Paulo, SP Brazil; 4 Department of Physiology and Biophysics, Institute of Biological Science, Federal University of Minas Gerais, Belo Horizonte, MG Brazil; 5 School of Veterinary, Department of Preventive Veterinary Medicine, Federal University of Minas Gerais, Av. Antônio Carlos 6627, Pampulha, Belo Horizonte, Minas Gerais 30161-970 Brazil

**Keywords:** Comparative proteomics, *Streptococcus agalactiae*, GBS, Genotypes, Fish, Human

## Abstract

**Background:**

*Streptococcus agalactiae* (GBS) is a major pathogen of Nile tilapia, a global commodity of the aquaculture sector. The aims of this study were to evaluate protein expression in the main genotypes of GBS isolated from diseased fishes in Brazil using a label-free shotgun nano-liquid chromatography-ultra definition mass spectrometry (nanoLC-UDMS^E^) approach and to compare the differential abundance of proteins identified in strains isolated from GBS-infected fishes and humans.

**Results:**

A total of 1070 protein clusters were identified by nanoLC-UDMS^E^ in 5 fish-adapted GBS strains belonging to sequence types ST-260 and ST-927 and the non-typeable (NT) lineage and 1 human GBS strain (ST-23). A total of 1065 protein clusters corresponded to the pan-proteome of fish-adapted GBS strains; 989 of these were identified in all fish-adapted GBS strains (core proteome), and 62 were shared by at least two strains (accessory proteome). Proteins involved in the stress response and in the regulation of gene expression, metabolism and virulence were detected, reflecting the adaptive ability of fish-adapted GBS strains in response to stressor factors that affect bacterial survival in the aquatic environment and bacterial survival and multiplication inside the host cell. Measurement of protein abundance among different hosts showed that 5 and 26 proteins were exclusively found in the human- and fish-adapted GBS strains, respectively; the proteins exclusively identified in fish isolates were mainly related to virulence factors. Furthermore, 215 and 269 proteins were up- and down-regulated, respectively, in the fish-adapted GBS strains in comparison to the human isolate.

**Conclusions:**

Our study showed that the core proteome of fish-adapted GBS strains is conserved and demonstrated high similarity of the proteins expressed by fish-adapted strains to the proteome of the human GBS strain. This high degree of proteome conservation of different STs suggests that, a monovalent vaccine may be effective against these variants.

**Electronic supplementary material:**

The online version of this article (10.1186/s12864-018-5423-1) contains supplementary material, which is available to authorized users.

## Background

*Streptococcus agalactiae* (Lancefield’s group B Streptococcus, GBS) is a major bacterial species of the genus *Streptococcus* and has medical and veterinary importance, affecting mainly humans [1, 2], cattle [3] and fish [4]. GBS is the most important pathogen of Nile tilapia, a global commodity of the aquaculture sector, causing outbreaks of septicemia and meningoencephalitis [4, 5].

The multilocus sequence typing (MLST) technique, which is considered the reference tool for genotyping GBS, allows the grouping of different strains according to the similarity of their allelic profiles (sequence typing – ST) and ancestry (clonal complex – CC) [6]. The strains belonging to CC1, CC17 and CC19 are generally human clinical isolates, whereas CC61 and CC67 consist exclusively of bovine isolates [7, 8]. The strains belonging to CC260, CC261, ST-257 and one non-typeable (NT) group lineage have been considered to be specialized for infect aquatic animal hosts [9]. These fish-adapted genotypic groups are genetically related based on the fact that their MLST profiles have been shown to share at least five identical alleles [9]. CC260 has been identified in GBS isolated from diseased fish in Brazil, Colombia, Costa Rica, Honduras and the USA [10–14], and CC261 has a worldwide distribution, having been detected in Israel, Australia, Belgium, the USA, Ghana, Indonesia and China [13–18], whereas the other genetic group composed of the ST-257 and NT strains occurs only in Brazil [9, 13]. In previous studies that classified seventy-five Brazilian GBS fish isolates into different MLST types, it was found that approximately 97% of the isolates belonged to the CC260 and NT strains [9, 10]. Considering the evolutionary relationship between these genotypes and the main GBS lineages that infect fishes in Brazil, it is necessary to understand the specific metabolic, adaptive and pathogenic characteristics of these genetic groups and their relationships to their aquatic hosts.

Proteomic studies make it possible to identify and quantify sets of proteins that are expressed by microorganisms under specific culture conditions [19]. Protein expression studies using GBS strains have highlighted the evaluation of surface proteins [20–23], secretory proteins [23, 24] and the comparative proteome [25]. These studies were conducted using isolates obtained from human [24] or fish hosts [23]; to date, no comparative proteomic studies of human and fish-adapted GBS strains or of GBS strains belonging to different genotypes have been performed. Pan-proteomics analysis, an alternative strategy that can be used to conduct comparative proteomic studies, seeks to compare the qualitative and quantitative proteome across strains, allowing interpretation of bacterial physiology and promoting knowledge of the genetic variation of each isolate [26]. Pan-proteomic analysis was previously used to determine the core and pan-proteome of four epidemic *Salmonella* Paratyphi A strains [19] and to compare the protein expression patterns of *Mycobacterium tuberculosis* strains with different virulence traits [27]. Thus, a pan-proteomic study of GBS strains that infect fishes would permit the analysis of protein variability within the strains belonging to the main Brazilian genotypes, increase scientific knowledge about the adaptation and pathogenesis of this bacterium in fishes, and make it possible to characterize its host-related adaptations. In addition, this approach would allow the identification of conserved antigenic proteins that can be used as targets in vaccine design.

This study aimed to evaluate the global abundance of proteins produced by the main genotypes of GBS isolated from fishes in Brazil using a label-free shotgun nano-liquid chromatography-ultra definition mass spectrometry (nanoLC-UDMS^E^) approach and to compare the differential expression of proteins identified in isolates obtained from human and fishes.

## Methods

### Bacterial strains

Five GBS strains previously isolated from diseased fish on different farms were selected from the National Reference Laboratory of Aquatic Animal Diseases (AQUACEN) culture collection and used in this study. These strains have whole-genome previously sequenced and belongs to different genotypes by MLST method [9]. SA16, SA20 and SA81 are from a group of NT strains, which have different genetic profiles determined according Godoy et al. [10] through of combination of MLST and the presence/absence of the genes *lmb*, *hylB* and *cylE*, and also from different fish hosts. SA53 is from ST-260 and SA95 is from ST-927. Additionally, the *S. agalactiae* NEM316 strain (ST-23), which was isolated from a human neonate with septicemia, was acquired from the American Type Culture Collection (strain designation ATCC12403) and included in this study to make it possible to compare the protein expression patterns of GBS strains isolated from fish and human hosts. The entire genome of the NEM316 strain has been sequenced and annotated (GenBank accession number NC_004368) [28]; its virulence genes have been well characterized, and several studies using transcriptomic and proteomic approaches have been conducted [24, 29–31]. Previous study from our group showed that NEM316 strain had the infection detected after 48 h post-inoculation, however the fish host did not show clinical signs and mortality on 15 days of challenger [32]. All strains were stored at − 70 °C until use. The characteristics of the strains are listed in Table 1.

**Table 1:** Characteristics of the *Streptococcus agalactiae* strains evaluated in this study

Isolate codes^a^	Host	Country/State	Year of isolation	Capsular serotype	ST	NCBI accession No.	Predicted Proteins / Protein clusters	Ref.
SA16	Nile tilapia	Brazil/São Paulo	2006	Ib	NT^b^	CP019807.1	1690/1652	[9]
SA20	Nile tilapia	Brazil/Paraná	2006	Ib	NT	CP003919.2	1679/1643	[9]
SA53	Nile tilapia	Brazil/Ceará	2007	Ib	260	CP019802.1	1700/1655	[9]
SA81	Amazon catfish	Brazil/Mato Grosso	2009	Ib	NT	CP019810.1	1687/1650	[9]
SA95	Nile tilapia	Brazil/Alagoas	2010	Ib	927	CP019812.1	1707/1662	[9]
NEM316	Human	Unknown	1975	III	23	NC_004368.1	2127/1968	[28]

^a^Strains refers to the identifier from AQUACEN culture collection

^b^Non-typeable

### Culture conditions

*S. agalactiae* strains isolated from fishes were thawed, streaked onto 5% sheep blood agar and incubated at 28 °C for 48 h according to the method described by Godoy et al. [10]. The NEM316 strain was incubated at 37 °C for 24 h according to the method described by Pereira et al. [32]. Each strain was inoculated into BHI broth (“Brain Heart Infusion”, Himedia, Mumbai, India) containing 0.05% (*v*/v) Tween 80 (BHIT) and cultured at 30 °C with gently agitation. Biological triplicate cultures of each strain were harvested for protein isolation upon reaching absorbances of 0.2 and 0.5 (OD_600_), equivalent to the mid-exponential phase of bacterial growth of the fish-adapted GBS strains (data not shown) and the NEM316 strain [30], respectively. The GBS strains were cultured under laboratory conditions at 30 °C; this corresponds to the temperature at which increased outbreaks of streptococcosis normally occur in fishes [4].

### Protein isolation

Extracts of the whole bacterial lysates from three biological replicates of each strain were prepared. The bacterial cells were harvested by centrifugation at 16,100 x g for 20 min at 4 °C. The bacterial pellets were washed three times with 10 mL of 50 mM Tris-HCl (pH 7.5) and collected by centrifugation after each wash. The bacterial pellets were then resuspended in 1 mL of lysis buffer (7 M urea, 2 M thiourea, 4% (*w*/*v*) CHAPS, 12.5 mM Tris-HCl and 1.5% (w/v) dithiothreitol (DTT) containing 10 μL of protease inhibitor mix (GE HealthCare, Pittsburgh, USA) and sonicated on ice using an ultrasonic cell disruptor (Unique, Indaiatuba, Brazil) for 20 min in cycles of 1 min at maximum power (495 W) followed by 1 min of rest. The lysates were centrifuged at 21,900 x g for 40 min at 4 °C; the supernatants were collected and subjected to five cycles of centrifugation at 15,000 x g for 30 min at 20 °C using Vivaspin 500 centrifugal concentrators (GE HealthCare) with a cutoff threshold of 3 kDa. Between cycles, the lysis buffer was exchanged for 50 mM ammonium bicarbonate (pH 8.5) to remove detergent from the samples. The extracted proteins were quantified using a Qubit 2.0 fluorometer (Invitrogen, Carlsbad, USA) and the Qubit protein assay kit (Molecular Probes, Oregon, USA) according to the manufacturer’s instructions.

### Protein digestion

A volume of 50 μL containing 2 μg.μL^− 1^ protein extract was collected from each replicate and transferred to a tube (1.5 mL) containing 10 μL of 50 mM ammonium bicarbonate. The proteins in the sample were denatured by the addition of 25 μL of 0.2% (*w*/*v*) *Rapi*GEST SF surfactant (Waters, Manchester, UK) at 80 °C for 15 min. Thiol groups were reduced using 2.5 μL of 100 mM DTT (Sigma Aldrich, Saint Louis, USA) at 60 °C for 30 min and alkylated using 2.5 μL of 300 mM iodoacetamide (Sigma Aldrich) at room temperature for 30 min in a dark chamber. The proteins in the sample were then enzymatically digested by addition of 5 μg of sequencing-grade modified trypsin (Promega, Madison, USA) and incubated at 37 °C for 16 h. Digestion was stopped by the addition of 10 μL of 5% (*v*/v) trifluoroacetic acid (Sigma Aldrich) and incubation at 37 °C for 90 min. The resulting peptide extracts were centrifuged at 21,900 x g for 30 min at 6 °C. The supernatants were collected, transferred to Waters Total Recovery vials (Waters), supplemented with 5 μL of 1 N ammonium hydroxide (Sigma Aldrich) and stored at − 70 °C until use.

### Mass spectrometry

Bidimensional nano ultra-performance liquid chromatography (nanoUPLC) tandem nano electrospray high-definition mass spectrometry (nanoESI-HDMS^E^) experiments were conducted using a 1-h reverse-phase (RP) gradient from 7 to 40% (v/v) acetonitrile (0.1% v/v formic acid) with a simulated 1D analysis and a delivery of 500 nL.min^− 1^ in a nanoACQUITY UPLC 2D Technology system (Waters). A nanoACQUITY UPLC High Strength Silica T3 column (1.8 μm, 100 μm × 10 cm, pH 3) was used in combination with an RP Acquity UPLC Nano Ease XBridge BEH130 C18 column (5 μm, 300 μm × 50 mm nanoflow column, pH 10). Typical on-column sample loads were 500 ng of total protein digest for each of the 5 fractions (500 ng/fraction/load).

For every measurement, the mass spectrometer was operated in resolution mode with a typical *m/z* resolving power of at least 25,000 full width at half-maximum (FWHM), an ion mobility cell that was filled with helium gas, and a cross-section resolving power of at least 40 Ω/Δ Ω. The effective resolution with the conjoined ion mobility was 25,000 FWHM. Analyses were performed using nano-electrospray ionization in positive ion mode nanoESI (+) and a NanoLock-Spray (both from Waters) ionization source. The lock mass channel was sampled every 30 s. The mass spectrometer was calibrated with the MS/MS spectrum of a solution of human [Glu^1^]-fibrinopeptide B (Glu-Fib) (100 fmol.μL^− 1^) that was delivered through the reference sprayer of the NanoLock-Spray source. The double-charged ion ([M + 2H]^2+^ = 785.8426) was used for initial single-point calibration, and MS/MS fragment ions of Glu-Fib were used to obtain the final instrument calibration.

Multiplexed data-independent acquisition (DIA) scanning with added specificity and selectivity conferred by a non-linear ‘T-wave’ ion mobility (HDMS^E^) device was performed on a Synapt G2-Si HDMS mass spectrometer (Waters). The spectrometer was automatically programmed to switch between standard MS (3 eV) and elevated collision energies HDMS^E^ (19–45 eV) applied to the transfer ‘T-wave’ collision-induced dissociation cell with nitrogen gas. The trap collision cell was adjusted to 1 eV using a millisecond scan time that was previously adjusted based on the linear velocity of the chromatographic peak that was delivered through a nanoACQUITY UPLC (Waters) to generate a minimum of 20 scan points for each single peak both in low-energy and high-energy transmission at an orthogonal acceleration time-of-flight (*oa*-TOF) and over a mass range of *m/z* 50 to 2000.

Mass spectrometric analysis of tryptic peptides was performed using a mass spectrometer equipped with a T-Wave-IMS device (Waters) in MS^E^ and UDMS^E^ modes according to the method previously described [33]. Stoichiometric measurements based on scouting runs of the integrated total ion account were performed prior to analysis to ensure standardized molar values across all samples. Based on these values, the tryptic peptides of each strain were injected onto the column in the same amounts. The radio frequency offset (MS profile) was adjusted such that the nanoESI-UDMS^E^ data were effectively acquired from *m/z* 400 to 2000 by MassLynx v.4.1 software (Waters), ensuring that any masses that were observed in the high-energy spectra with less than *m/z* 400 arose from dissociations in the collision cell. The MS proteomics data are available at the ProteomeXchange Consortium via the PRIDE [34] partner repository under the identifier PXD008744.

### Protein identification and quantification

The UDMS^E^ raw data were processed using Progenesis QI for Proteomics (QIP) v.2.0 (Nonlinear Dynamics, Newcastle, UK) according to the method previously described by Kuharev et al. [35]. Imported runs were subjected to automatic data processing for protein identification and quantitative information using the following parameters: peak picking limits = 5 and maximum charge retention time limits = 8.

An *in-house* database was created using protein code sequences (CDSs) of the whole genomes of the strains obtained from the GenBank database [36]. CD-HIT software version 4.6 [37] was used with the -c parameter equal to 1 to create a non-redundant set of CDSs according to the recommendation of Broadbent et al. [26]. The database management tool of the ProteinLynx Global Server (PLGS) v 3.0.2 (Waters) was used to append reversed sequences (to assess the false positive rate during identification) and to create the final fasta file of the used database.

The following parameters were used for peptide identification: digest reagent = trypsin; maximum missed cleavages = 1; maximum protein mass = 600 kDa; modifications: carbamidomethyl of cysteine (fixed), acetyl N-terminal (variable), phosphoryl (variable), and oxidation of methionine (variable); search tolerance parameters: peptide tolerance = 10 ppm, fragment tolerance = 20 ppm, and maximum false discovery rate (FDR) = 4%. Ion matching requirements used the default parameters [38], which were fragments per peptide = 1, fragments per protein = 3, and peptides per protein = 1. The protein-level quantitation was performed with relative quantitation using the Hi-N algorithm, which is incorporated in Progenesis QIP. Peptides with scores ≤3, mass errors ≥20 ppm, or sequence length ≤ 6 amino acids and those found in the decoy reverse database were removed. Proteins identified on the basis of at least two peptides (with ≥1 proteotypic peptide per protein) and that were present in ≥2 of the three biological replicates for each GBS strain were considered.

The variability and quality of the proteomic data were analyzed through principal component analysis (PCA), distribution of peptide precursors and fragment error, peptide match distribution, drift time, number of times that an identified protein appeared in the biological replicates and dynamic range. The PCA biplot was generated using the *ggbiplot* package version 0.55 [39] in R software version 3.4.1 [40]; the other plots were generated from fragment, peptide and protein tables obtained during searching of parameters for peptide identification using TIBCO SpotFire software version 7.0 (TIBCO, Boston, USA). The dynamic range of protein amounts of the identified proteins from all strains was calculated using the average relative abundance of each biological triplicate against protein rank. The data were binned by log_10_ of their normalized abundance, ordered in decreasing sequence and plotted using TIBCO SpotFire software.

The Progenesis QIP algorithm was used to organize the identified proteins into a list of proteins with statistically significant differences in expression (ANOVA, *p-value* ≤ 0.05). A protein was considered to be differentially expressed with respect to NEM316 if there was a ≥ 2-fold change in its expression (log_2_ ratio ≥ 1 for proteins with higher abundance levels or log_2_ ratio ≤ − 1 for proteins with lower abundance levels). A heat map was generated from normalization of the log_2_ value of each protein by *z*-score calculation. Clustergrams were created using the unweighted pair group method with the average (UPGMA) approach and Euclidean distance in TIBCO SpotFire software. A similarity matrix was generated according to the agreement between identified proteins of each strain and visualized using the *gplots* package version 3.0.1 in R software [41].

### Bioinformatics analysis

The Interactivenn web-based tool [42] was used to evaluate the number of proteins identified in each GBS strain through Venn diagrams. The core proteome consisted of the subset of identified proteins in all evaluated strains. The accessory proteome consisted of the subset of identified proteins shared between at least two strains, and the unique proteome consisted of the proteins that were identified exclusively in a particular strain.

Predicted protein clustering (PPC) to indicate the homologous genes between the strains was performed using OrthoMCL software [43] using the default parameters. In summary, files containing the CDS of each whole-genome were concatenated and adjusted using OrthoMCL scripts. A BLASTp analysis was applied to the resulting concatenated file against itself using an e-value of 10e-20.

To predict orthologous groups by functional category and subcellular localization, the sequences of identified proteins were analyzed using the Cluster of Orthologous Genes (COG) database version 2014db [44] and SurfG+ software version 1.0.2 [45], respectively. The COG database search was performed using an *in-house* script (available at https://github.com/aquacen/blast_cog).

The protein-protein interaction network of identified proteins in the core proteome was built using the STRING tool version 10.5 [46] using the *S. agalactiae* NEM316 strain as the reference and the following settings: meaning of network edges = confidence; active interaction sources = experiments, gene fusion, databases, co-occurrence and co-expression; and minimum required interaction score = 0.980. Predicted interactions were tested for enrichment for Kyoto Encyclopedia of Genes and Genomes (KEGG) pathway maps in STRING.

Prediction of vaccine candidates was performed using the Vaxign webserver [47]. Dynamic Vaxign analysis was performed with the CDS of identified proteins of the core proteome with the subcellular localizations Secreted, Potentially surface-exposed (PSE) or Membrane from SurfG+ analysis. Parameters of gram-positive bacterium and similarity to host proteins of humans were set. Only proteins with adhesion probabilities ≥0.51 were included in the result.

## Results

### Label-free proteomics results

The proteomes of GBS strains isolated from fishes (*n* = 5) and human (*n* = 1) were determined by LC-UDMS^E^. A total of 32,145 peptides with a normal distribution of 10 ppm error (~ 92% of the peptides were detected with an error of less than 10 ppm) were identified (Additional file 1: Figure S1A). Approximately 44% of the peptides were identified from peptide match type data in the first pass (PepFrag1), whereas ~ 15% were obtained in the second pass (PepFrag2); 7, 7% and ~ 26% of the peptides were identified as missed trypsin cleavage, in-source fragmentation and variable modifications (VarMod), respectively (Additional file 1: Figure S1B). Of the total peptides, ~ 87% showed a charge state of at least [M + 2H]^2+^ (Additional file 1: Figure S1C), and 90–97% of the identified proteins were found in 3 of the 3 replicates of each GBS strain (Additional file 1: Figure S1D). The PCA analysis clustered the GBS isolates based on the proximity of points identified for each strain, demonstrating the reproducibility of the proteomic data among the replicates (Additional file 1: Figure S1E).

The total number of identified proteins in each biological replicate of the individual strains varied from 1216 to 1247, resulting in the detection of 1273 proteins in at least two of the three replicates. An average of 25 peptides per protein and an FDR of 0.08% when decoy detection was set at agreement of two of the three replicates were found for each strain. To avoid overestimation of the number of identified proteins based on the pan-proteome database used, protein clustering of highly homologous sequences was performed. The PPC of the genome sequences of the six strains was 2148 (predicted pan-proteome), and the identified proteome was composed of 1070 protein clusters. The proteins and clusters identified in this study are shown in Additional file 2: Table S1.

### Pan-proteome of fish-pathogenic *S. agalactiae* strains

**Fig. 1:**
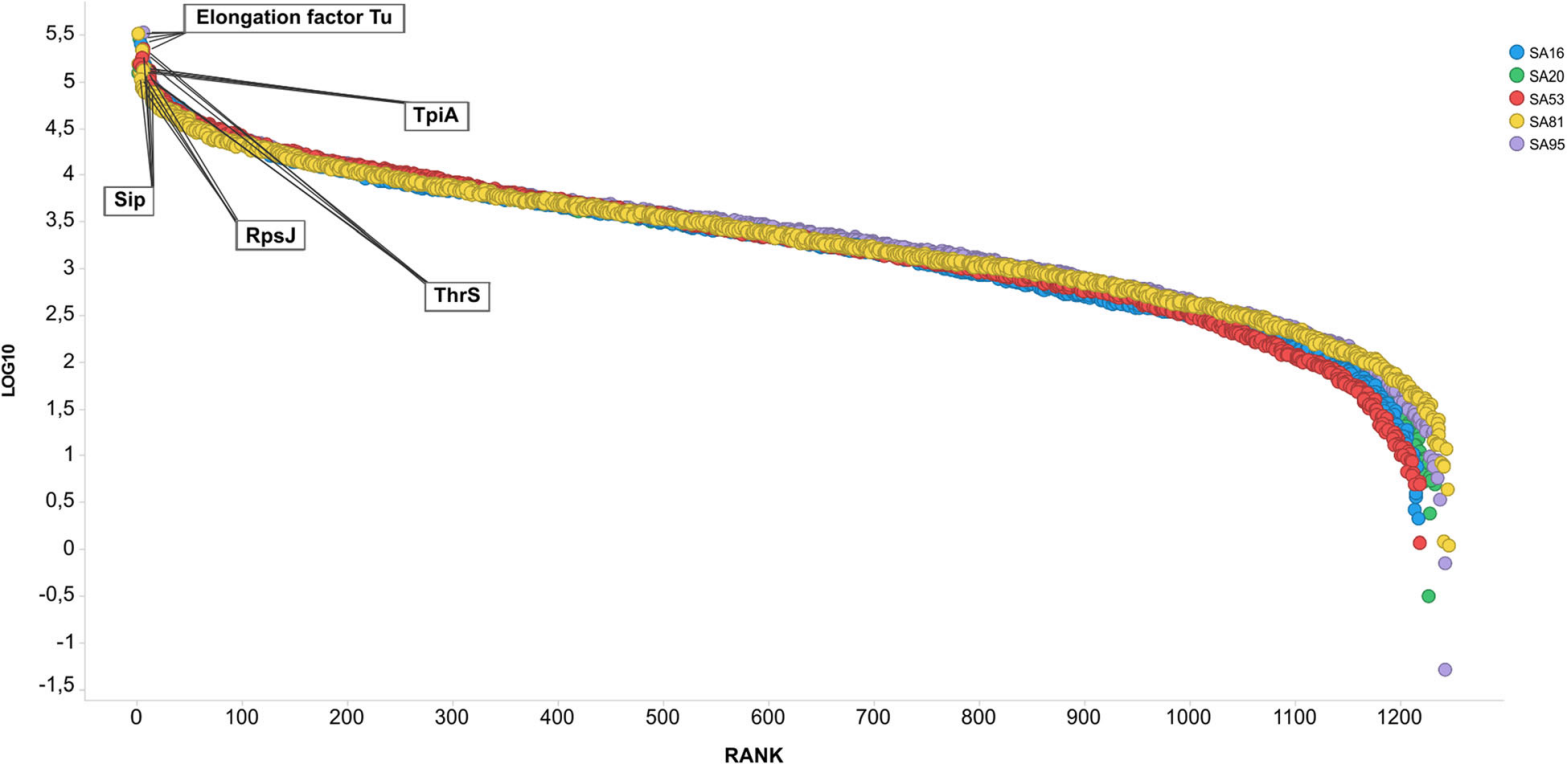
Dynamic range of the protein abundances found in fish-adapted GBS strains. The data are binned according to the log10 of their normalized abundance. SA16 (blue), SA20 (green), SA53 (red), SA81 (yellow), and SA95 (purple)

A total of 1020 proteins were identified in SA16, corresponding to ~ 62% of the strain’s PPC; 1036 proteins were identified in SA20 (~ 63% of the strain’s PPC), 1023 proteins were identified in SA53 (~ 62% of the strain’s PPC), 1051 proteins were identified in SA81 (~ 64% of the strain’s PPC) and 1040 proteins were identified in SA95 (~ 63% of the strain’s PPC). The dynamic range of the quantified proteins of GBS between the most and least abundant proteins in all strains was ~ 5 log units. The most abundant proteins identified were associated with virulence, metabolism and regulation. Elongation factor Tu, TpiA, RpsJ, Sip and ThrS were among the top 10 most abundant proteins identified in all fish-adapted GBS strains (Fig. 1).

**Fig. 2:**
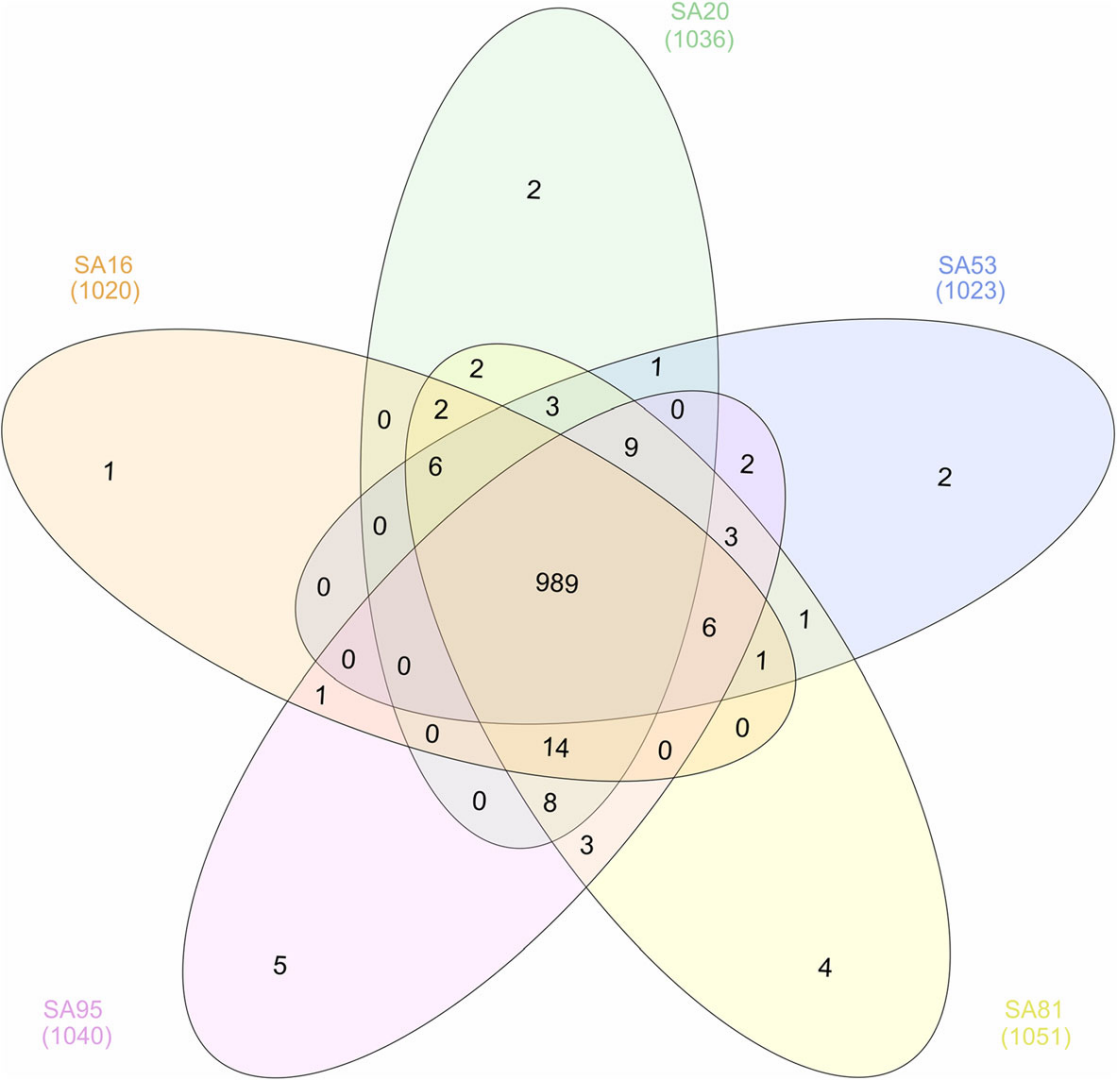
Venn diagram showing the number of unique and shared proteins in fish-adapted GBS strains

**Fig. 3:**
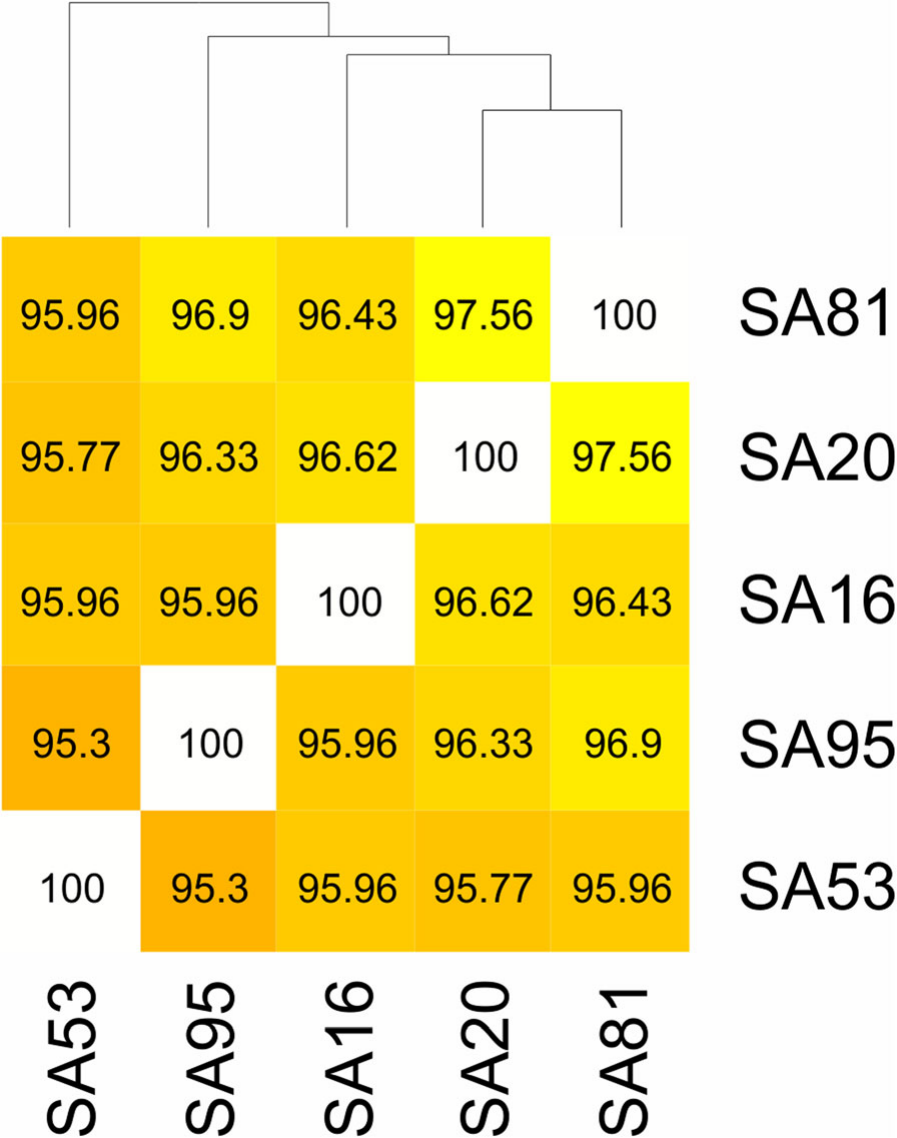
Proteome similarity matrix of fish-adapted GBS strains. The numbers inside the frames indicate the percentage of similarities among strains

To investigate the proteome shared by the isolates, a comparative analysis of the identified proteins in each strain was performed using Venn diagrams. A total of 989 proteins were identified in the core proteome, 62 proteins were present in the accessory proteome, and 1, 2, 2, 4 and 5 proteins were exclusively identified in SA16, SA20, SA53, SA81 and SA95, respectively (Fig. 2 and Additional file 2: Table S1). Therefore, the identification of 1065 proteins corresponds to a pan-proteome that is representative of the evaluated fish-adapted GBS strains. The fish-adapted GBS strains were closely related (similarity > 95.3%) in protein content (Fig. 3) even among strains isolated from different fish species, considering that SA81 was isolated from Amazon catfish, whereas the other fish-adapted GBS strains were obtained from diseased Nile tilapia. The core proteome represented 92.42% of the expressed pan-proteome, suggesting that protein expression is conserved among fish-adapted GBS strains.

**Fig. 4:**
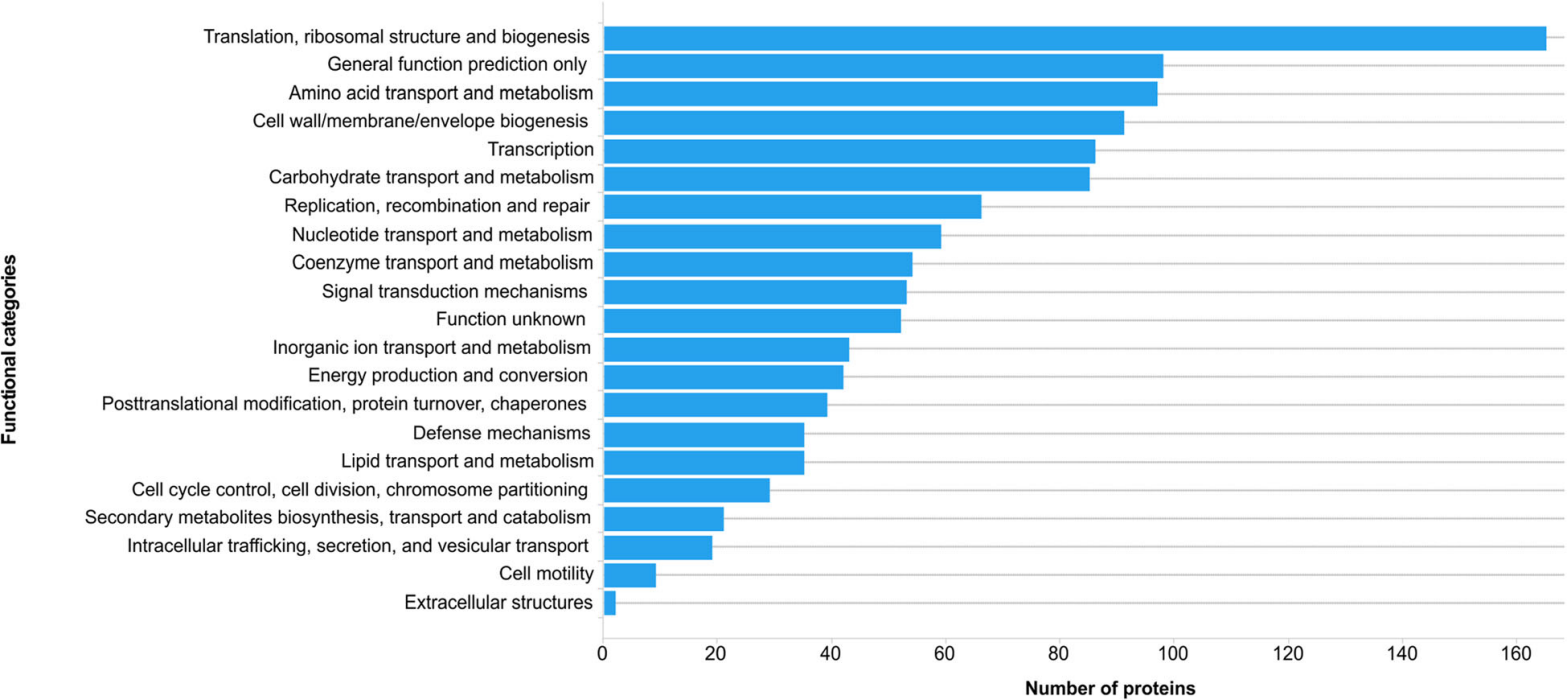
Prediction of COG functional categories of the proteins identified in the pan-proteome of fish-adapted GBS strains. The total number of proteins in the functional categories does not correspond to the final number of proteins in the pan-proteome because some proteins are associated with more than one COG category

Approximately 95% of the pan-proteome (*n* = 1018) of the fish-adapted GBS strains was classified into 20 functional categories using COG; the remaining ~ 5% of the identified proteins (*n* = 52) were classified as having unknown functions. The most common categories were translation/ribosomal structure and biogenesis (*n* = 165), general function prediction only (*n* = 98), amino acid transport and metabolism (*n* = 97), cell wall/membrane/envelope biogenesis (*n* = 91), transcription (*n* = 86) and carbohydrate transport and metabolism (*n* = 85) (Fig. 4). The main proteins detected in each functional category are shown in Table 2. In addition, proteins related to the uptake of amino acids, carbohydrate and metallic ions as ABC transporters and phosphoenolpyruvate-dependent phosphotransferase (PTS) systems were also identified.

**Table 2:** Main proteins identified in the pan-proteome of fish-adapted GBS strains and their classification into different COG functional categories

COG Category	Function	Proteins
Cellular processes and signaling		
Cell cycle control, cell division, chromosome partitioning	Cell division	FtsL, FtsE, FtsZ and DivIVA
Cell wall/membrane/envelope biogenesis	Capsular polysaccharide biosynthesis	CpsB, CpsC, CpsD, CpsG, NeuB, NeuC
	Peptidoglycan biosynthesis	MurA, MurD, MurE, MurF, MurG
	Group B antigen	RmlA, RmlB, RmlC
	Multidrug resistance	PbpX, Pbp1A, Pbp2A, DltB
	Immunoreactive antigen	PcsB, Sip
Cell motility	Transport	Asp1, CglA
Post-translational modification, protein turnover, and chaperones	Heat shock	GroL, GroS, GrpE, DnaK, DnaJ, CplX, CplP, HslO
	Oxidative stress resistance	Trx, TrxB, Thioredoxin, SufB, SufC
Signal transduction mechanisms	Two-component system	CiaR, CovS/CovR, Stp1/Stk1
	Stress response	Universal stress protein
Intracellular trafficking, secretion, and vesicular transport	Protein translocation	SecA, SecY
Defense mechanisms	Multidrug resistance	EcsA, Beta-lactamase
Information storage and processing		
Translation, ribosomal structure and biogenesis	30S ribosomal proteins	RpsJ, RpsS, RpsC, RpsQ, RpsH, RpsE, RpsM, RpsK, RpsO, RpsI, RpsT, RpsA, RpsP, RpsU, RpsR, RpsF, RpsN, RpsG, RpsL, RpsB, RpsD
	50S ribosomal proteins	RplC, RplD, RplW, RplB, RplV, RplP, RplN, RplX, RplE, RplF, RplR, RplO, RplQ, RplM, RplS, RplU, RplT, RplA, RplK, RpmC, RpmD, RpmB, RpmL, RpmG
Transcription	RNA polymerase	RpoA, RpoB, RpoC, RpoE, RpoZ
	Transcriptional regulators	MutR, GntR, LysR, LacI, MarR, MerR, DeoR, TetR
Replication, recombination and repair	DNA replication	DnaA, DnaN, GyrA, GyrB
	DNA mismatch repair	MutL, MutS
Metabolism		
Energy production and conversion	Proton transporting	AtpA, AtpC, AtpD, AtpF, AtpG
	Pyruvate metabolism	pdhC, TPP-dependent acetoin dehydrogenase complex, Branched-chain alpha-keto acid dehydrogenase subunit E2, Dihydrolipoyl dehydrogenase
Amino acid transport and metabolism	Arginine metabolism	ArcA, ArcB, ArcC, ArcD, ArgF, ArgG, ArgH, ArgR, ArgS
	Threonine metabolism	Hom, ThrB, ThrC
	Glycine metabolism	GlyA
	Glutamic acid metabolism	AlaT
	Glutamine metabolism	GlnA
	Serine metabolism	SdhA, SdhB, SerB, SerC
	Aspartic acid metabolism	AsnA
	Cysteine metabolism	Cysteine desulfurase
	Methionine metabolism	MetK, MetN
Nucleotide transport and metabolism	Purine biosynthesis	GuaC, PurA, PurB, PurC, PurD, PurE, PurF, PurH, PurK, PurM, PurN, PurR
	Pyrimidine biosynthesis	PyrC, PyrD, PyrE, PyrG, PyrH, PyrR
Carbohydrate transport and metabolism	Glycogen synthesis	PgmA
	Glycolytic pathway	Eno, Pga, Pgk, TpiA
	Pentose phosphate pathway	AroD
	PTS system	12 proteins involved in transport of ascorbate, glucose, beta-glucoside, lactose, mannose, galactitol and fructose
Coenzyme transport and metabolism	Riboflavin biosynthesis	RibBA, RibD, RibE, RibH
Lipid transport and metabolism	Fatty acid biosynthesis	AccD, FabD, FabF, FabG, FabH, FabT, FabZ
Inorganic ion transport and metabolism	Transporter	Iron, Nickel, Ferrichrome, Manganese, Magnesium, Potassium, Phosphate, Heme
	Zinc metabolism	Zinc-binding protein
	Copper metabolism	CutC
Secondary metabolites biosynthesis, transport, and catabolism	Multidrug resistance	DltA, Bleomycin resistance protein
Poorly characterized		
General function prediction only	Phenazine biosynthesis	PhzF
Mobilome	Prophages	Phage repressor protein
	Transposons	Transposase
Function unknown	Stress response	Gls24, General stress protein

According to the subcellular localization analysis, the identified proteins in the core proteome of fish-adapted GBS strains included 880 cytoplasmic proteins, 99 PSE, 57 membrane proteins and 29 secreted proteins. A total of 166 non-cytoplasmic proteins were evaluated as putative vaccine targets, and 38 of these showed an adhesion probability ≥0.51 (Table 3). Bacterial virulence proteins related to adhesion, invasion, immune evasion and resistance to cationic antimicrobial peptides were found in fish-adapted GBS strains (Table 4).

**Table 3:** Putative vaccine targets for fish-adapted GBS strains identified by reverse vaccinology strategy using the expressed core proteome

Accession	Cluster	Protein	Adhesin Probability
SaSA20_0144	Cluster0008	ABC transporter substrate-binding protein	0.552
SaSA20_0016	Cluster0032	PcsB protein	0.746
SaSA20_0031	Cluster0046	sip Group B streptococcal surface immunogenic protein	0.695
SaSA20_0155	Cluster0160	Membrane protein	0.545
SaSA20_0222	Cluster0210	Glycine/betaine ABC transporter substrate-binding protein	0.536
SaSA20_0262	Cluster0242	Amino acid ABC transporter substrate-binding protein	0.521
SaSA20_0270	Cluster0249	Penicillin-binding protein 1A	0.699
SaSA20_0443	Cluster0363	Peptidase S16	0.548
SaSA20_0646	Cluster0517	Cell wall surface anchor protein	0.548
SaSA20_0682	Cluster0548	Foldase protein PrsA	0.613
SaSA20_0761	Cluster0612	Hypothetical protein	0.586
SaSA20_0810	Cluster0648	Hypothetical protein	0.561
SaSA20_0950	Cluster0759	Hypothetical protein	0.581
SaSA20_0971	Cluster0779	Hypothetical protein	0.541
SaSA20_1003	Cluster0802	Zn-dependent protease	0.666
SaSA20_1090	Cluster0876	Choline binding protein D	0.538
SaSA20_1101	Cluster0885	ABC transporter substrate-binding protein	0.594
SaSA20_1126	Cluster0908	Membrane protein	0.638
SaSA20_1161	Cluster0932	Hypothetical protein	0.576
SaSA20_1201	Cluster0960	Glutamine ABC transporter permease	0.614
SaSA20_1259	Cluster1012	Manganese ABC transporter substrate-binding protein	0.570
SaSA20_1283	Cluster1027	Acyltransferase	0.540
SaSA20_1328	Cluster1069	ABC transporter substrate-binding protein	0.562
SaSA20_1357	Cluster1094	Lipoprotein	0.528
SaSA20_1358	Cluster1095	Amino acid ABC transporter substrate-binding protein	0.575
SaSA20_1625	Cluster1313	Phosphate-binding protein PstS 2	0.530
SaSA20_1659	Cluster1339	cAMP factor	0.610
SaSA20_1679	Cluster1357	Hypothetical protein	0.553
SaSA20_1731	Cluster1400	Membrane protein	0.669
SaSA20_1745	Cluster1413	Peptidoglycan-binding protein LysM	0.713
GBS_RS02350	Cluster1590	Cell wall surface anchor protein	0.573
SaSA20_0112	Cluster1592	rbsB D-ribose-binding protein	0.568
SaSA16_1063	Cluster1614	Hypothetical protein	0.571
SaSA53_1173	Cluster1726	fhuD Ferrichrome ABC transporter substrate-binding protein	0.527
GBS_RS11195	Cluster1752	Cell surface protein	0.527
GBS_RS03585	Cluster1754	Cell wall surface anchor protein	0.604
GBS_RS03520	Cluster1888	Acid phosphatase/phosphotransferase	0.596
GBS_RS05110	Cluster1920	BMP family ABC transporter substrate-binding protein	0.597

**Table 4:** Proteins potentially involved in virulence identified in the pan-proteome of fish-adapted GBS strains

Accession	Description	H^a^	F^b^	Function	Reference^c^
Adhesion					
SaSA20_0637	Elongation factor Tu	X	X	Mediate bacterium binding to fibronectin, fibrinogen and mucin	[76–78]
SaSA20_0646	Cell wall surface anchor protein	X	X	Cell adhesion	[23]
SaSA20_0697	gapN Glyceraldehyde-3-phosphate dehydrogenase	X		GBS adhesion to extracellular matrix and cytoskeletal proteins of host cells	[101]
SaSA20_1009	Dihydroorotate dehydrogenase (PavA)	X		Interacts with cell surface fibronectin	[102]
SaSA20_1475	gap Glyceraldehyde-3-phosphate dehydrogenase	X	X	GBS adhesion to extracellular matrix and cytoskeletal proteins of host cells	[101]
SaSA20_1675	Hypothetical protein (BibA)	X	X	GBS adhesion to human epithelial cells and binding to complement regulatory protein C4bp, acting as anti-phagocytic factor	[74]
Evasion					
GBS_RS07805	Nucleotide sugar dehydratase	X	X	Prevents deposition of complement factor C3b and inhibits the opsonophagocytosis	[28]
SaSA16_1205	Family 2 glycosyltransferase			Acts in immune recognition, bacterial evasion, intercellular signaling and biofilm formation	[85]
SaSA20_0016	PcsB protein	X		Peptidoglycan hydrolase	[23]
SaSA20_0031	sip Group B streptococcal surface immunogenic protein	X	X	Protective antigen and vaccine target	[93]
SaSA20_0382	Reticulocyte binding protein	X		Carbohydrate binding	[23]
SaSA20_0980	neuA N-acylneuraminate cytidylyltransferase	X		Prevents deposition of complement factor C3b and inhibits the opsonophagocytosis	[28]
SaSA20_0981	NeuD protein	X		Prevents deposition of complement factor C3b and inhibits the opsonophagocytosis	[28]
SaSA20_0982	UDP-N-acetylglucosamine-2-epimerase NeuC	X		Prevents deposition of complement factor C3b and inhibits the opsonophagocytosis	[28]
SaSA20_0983	N-acetyl neuramic acid synthetase NeuB	X		Prevents deposition of complement factor C3b and inhibits the opsonophagocytosis	[28]
SaSA20_0987	Capsular polysaccharide biosynthesis protein	X		Prevents deposition of complement factor C3b and inhibits the opsonophagocytosis	[28]
SaSA20_0990	UDP-N-acetylglucosamine:LPS N-acetylglucosamine transferase	X	X	Prevents deposition of complement factor C3b and inhibits the opsonophagocytosis	[28]
SaSA20_0992	Tyrosine-protein kinase CpsD	X	X	Prevents deposition of complement factor C3b and inhibits the opsonophagocytosis	[28]
SaSA20_0993	Capsular polysaccharide biosynthesis protein CpsC	X		Prevents deposition of complement factor C3b and inhibits the opsonophagocytosis	[28]
SaSA20_0994	Tyrosine-protein phosphatase CpsB	X		Prevents deposition of complement factor C3b and inhibits the opsonophagocytosis	[28]
SaSA20_1017	rmlB dTDP-glucose 4,6-dehydratase	X		Biosynthesis of group B antigen	[28]
SaSA20_1018	rmlC dTDP-4-dehydrorhamnose 3,5-epimerase	X		Biosynthesis of group B antigen	[28]
SaSA20_1019	rmlA Glucose-1-phosphate thymidylyltransferase	X		Biosynthesis of group B antigen	[28]
SaSA20_1438	Peptidase	X	X	Prevention of complement factor C5a deposition and phagocytosis from host cells	[57]
Invasin					
GBS_RS03175	TlyA family rRNA (cytidine-2’-O)-methyltransferase	X		Hemolytic activity in different pathogenic bacteria	[103]
SaSA20_0534	eno Enolase	X	X	Binding or activation of plasminogen by GBS during incubation in human blood	[29]
SaSA20_0586	Glycosyl transferase family 1 (IagA)	X	X	GBS blood-brain barrier penetration in neonates	[75]
SaSA20_1659	cAMP fator	X	X	Forms pore on membrane of the host cells	[57]
SaSA53_0799	Internalin			Required by *Listeria monocytogenes* for invasion of different nonphagocytic mammalian cell lines	[104]
Multidrug Resistance					
SaSA20_0259	pbpX Penicillin-binding protein 2x	X		Mediate GBS resistance to AMPs	[57]
SaSA20_0270	Penicillin-binding protein 1A	X		Mediate GBS resistance to AMPs	[57]
SaSA20_1495	D-alanyl-lipoteichoic acid biosynthesis protein DltD	X	X	Resist AMPs is to decrease the charge on their cell surface	[57]
SaSA20_1499	dltA D-alanine--poly(phosphoribitol) ligase subunit 1	X	X	Resist AMPs is to decrease the charge on their cell surface	[57]
SaSA20_1677	pbp2A Penicillin-binding protein 2A	X		Mediate GBS resistance to AMPs	[57]

^a^The letter X in this column represents that the virulence protein was previously identified in GBS human strains

^b^The letter X in this column represents that the virulence protein was previously identified in GBS fish strains

^c^Reference based on function of each virulence protein

### Interactome analysis of the core proteome

**Fig. 5:**
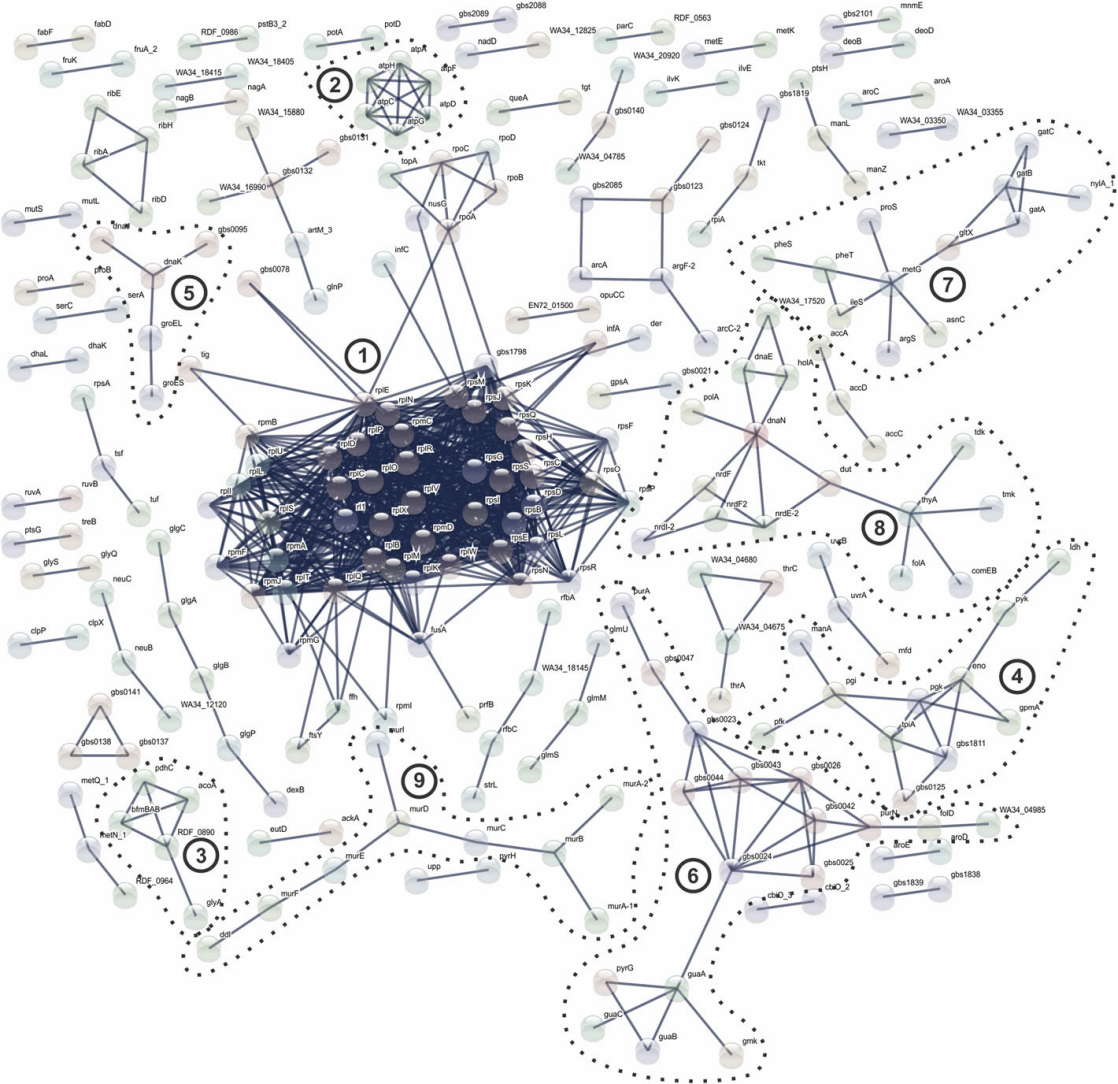
Interaction networks of identified core proteins of fish-adapted GBS strains. Thicker lines denote interactions with score ≥ 0.980. (1) Ribosomal proteins; (2) ATP synthase; (3) Pyruvate metabolism; (4) Carbohydrate metabolism; (5) Heat shock proteins; (6) Nucleotide metabolism; (7) Aminoacyl-tRNA synthetase; (8) DNA replication and repair; (9) Peptidoglycan biosynthesis

To better understand the biological functions of the proteins in the core proteome of fish-adapted GBS strains, a protein-protein interaction (PPI) analysis was conducted. As shown in Fig. 5, the greatest numbers of interactions were verified in the proteins related to ribosomal proteins (cluster 1; *n* = 62), ATP synthase (cluster 2; *n* = 6), pyruvate metabolism (cluster 3; *n* = 5), carbohydrate metabolism (cluster 4; *n* = 11), heat shock proteins (cluster 5; *n* = 5), nucleotide metabolism (cluster 6; *n* = 17), aminoacyl-tRNA synthetase (cluster 7; *n* = 12), DNA replication and repair (cluster 8; *n* = 15) and peptidoglycan biosynthesis (cluster 9; *n* = 9).

To determine the metabolic network of GBS that infect fishes, the proteins identified in the core proteome were analyzed using pathway enrichment analysis. The results revealed that a total of 28 pathways showed significant values (FDR < 0.05); the pathways most highly related to the dataset were metabolic pathways (FDR < 6.88e-36), biosynthesis of secondary metabolites (FDR < 2.37e-23), microbial metabolism in diverse environments (FDR < 1.47e-9), and ribosome (FDR < 3.82e-16) (Additional file 3: Table S2).

### Differential expression of proteins among GBS strains

To evaluate the abundance of specific proteins in strains of different host origins, a comparative analysis of the proteome of the NEM316 strain (isolated from a human) and the fish-adapted GBS strains was performed. A total of 1044 proteins were identified in the NEM316 strain, corresponding to ~ 53% of the proteins identified in PPC. Five and 26 proteins were exclusively expressed in the NEM316 strain and the fish-adapted GBS strains, respectively (Additional file 4: Table S3). The proteins exclusively expressed in the NEM316 strain are involved in transcription (*n* = 1), cell wall/membrane/envelope biogenesis (*n* = 1), nucleotide metabolism (*n* = 1), posttranslational modification, protein turnover, chaperones (*n* = 1) and unknown functions (*n* = 1). On the other hand, the proteins exclusively identified in the fish-adapted GBS strains are involved in putative multidrug resistance (D-alanyl-D-alanine carboxypeptidase, bacteriocin transport accessory protein, bleomycin resistance protein and Pbp2B), hemolysin (cAMP factor), evasins (CpsG and NeuB) and host colonization (Type VII secretion protein EsaA). Two proteins involved in oxidative stress resistance (flavoprotein and phenazine biosynthesis protein) were also identified. Of the identified proteins, five are involved in metabolism (PTS mannose transporter subunit IIB, beta-hexosamidase, 5-formyltetrahydrofolate cyclo-ligase, malate dehydrogenase, gluconate 5-dehydrogenase), two are involved in information storage and processing (ribonuclease HII and 3′-5′ exoribonuclease), three are involved in cellular processes and signaling (PhoB, Asp1 and ATPase AAA), and six are poorly characterized (five hypothetical proteins and a membrane protein).

The subset of core proteins decreased slightly from 989 to 978 proteins with the addition of the proteome of the NEM316 strain, and the pan-proteome increased to 1070 proteins. This strain showed similarity of protein content to that of the fish-adapted GBS strains of 94.3 to 97.3% (data not shown).

**Fig. 6:**
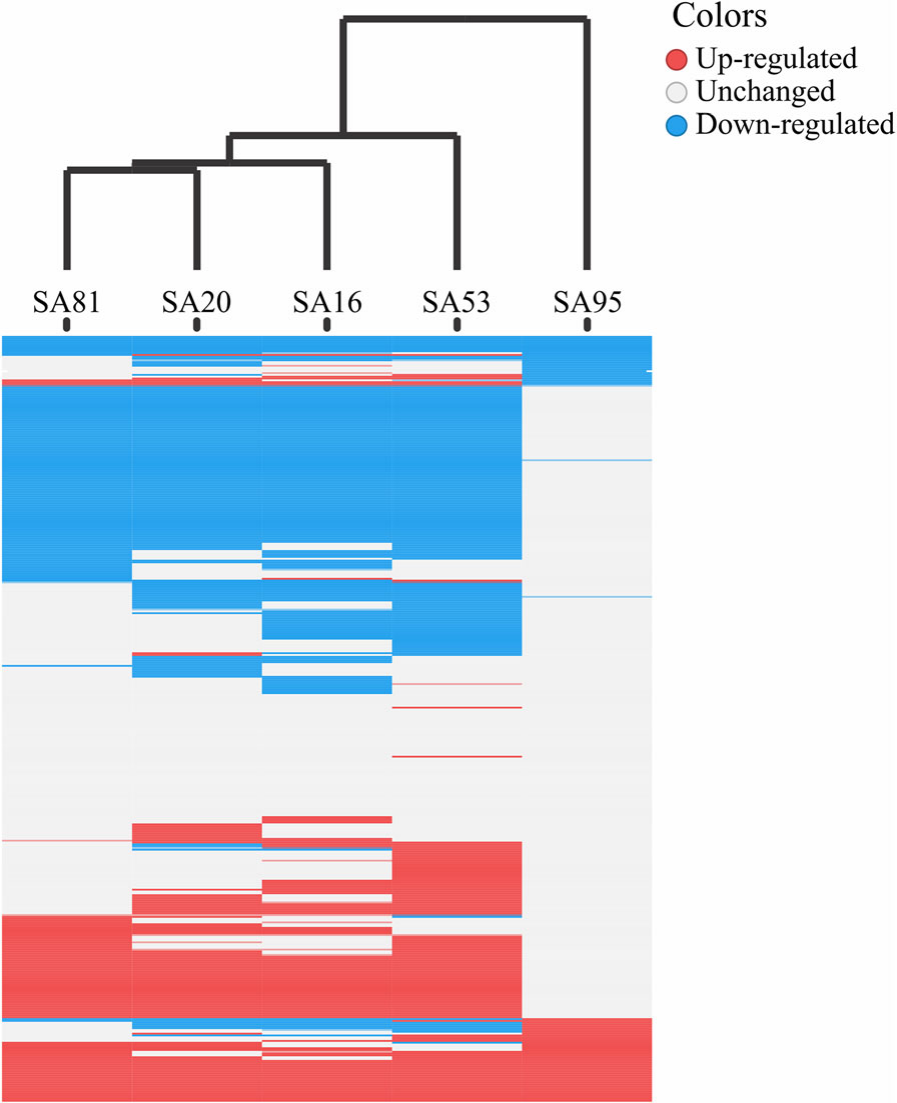
Heat map analysis of proteins that were significantly up- and down-regulated in fish-adapted GBS strains in comparison to the NEM316 strain

**Fig. 7:**
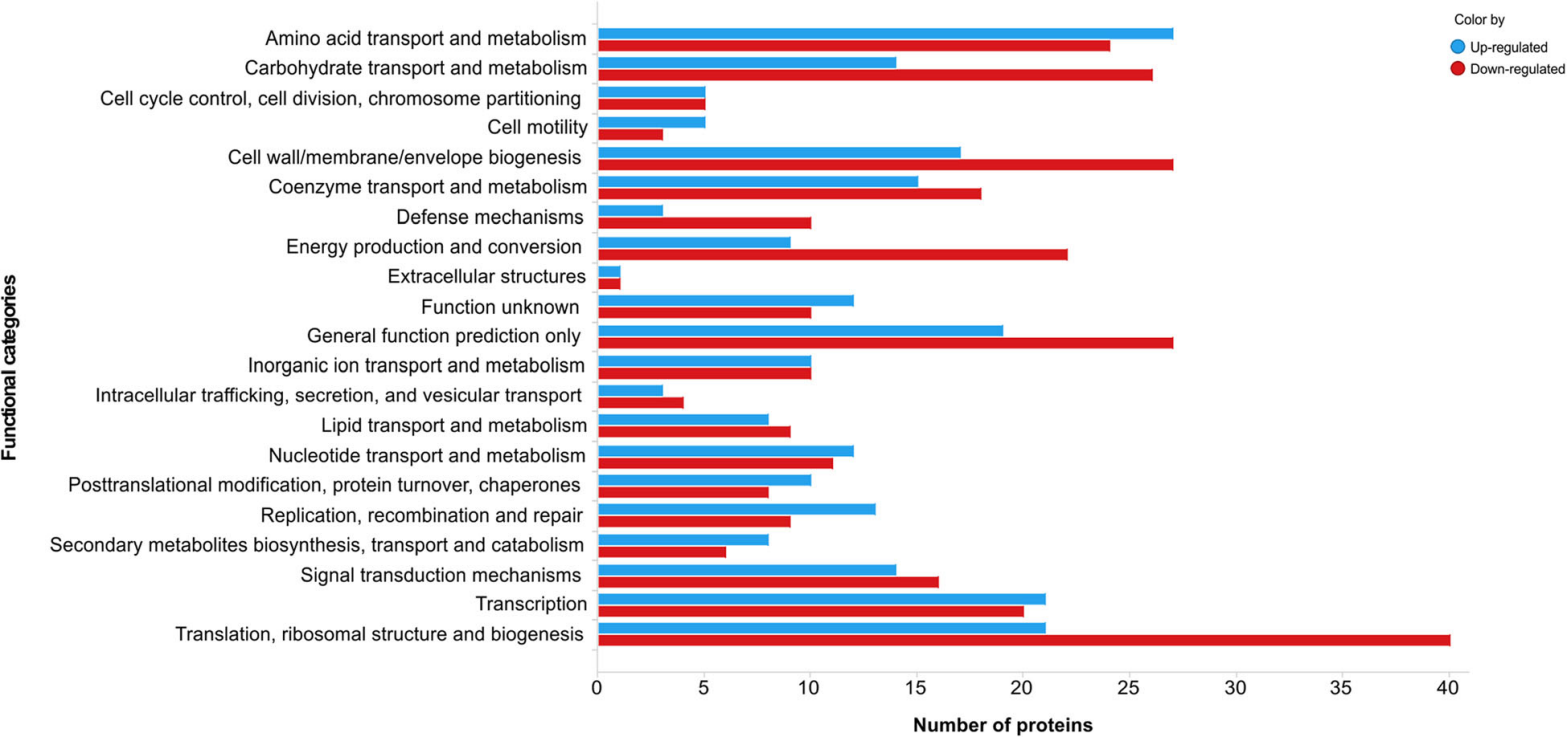
COG functional categories of the proteins that were differentially expressed in human- and fish-adapted GBS strains. The blue bars represent up-regulated proteins in fish-adapted GBS strains with respect to the NEM316 strain; the red bars represent down-regulated proteins

In the expression analysis, only proteins with *p* ≤ 0.05 and common to the six strains were considered (*n* = 534). The numbers of differentially expressed proteins (DEPs) in each strain are presented in Additional file 5: Table S4. In comparison to the other strains, the NEM316 and SA95 strains are closely related to each other, as shown by the lower number of DEPs between them (*n* = 93). In the NT strains, an average of 307 proteins varied in expression by 2-fold from their expression in the NEM316 strain. The highest variation in the number of DEPs was detected between SA53 and NEM316 (*n* = 358). A total of 215 and 269 proteins were up- and down-regulated, respectively, in fish-adapted GBS strains compared to the human GBS strain (Additional file 6: Figure S2 and Additional file 7: Table S5). Of these, 29 and 11 proteins were identified as up- and down-regulated, respectively, in all fish-adapted GBS strains (Additional file 6: Figure S2 and Table 5). A hierarchical clustering analysis was performed, and the results revealed an association between the regulatory level of proteins and the genotypes of the tested fish-adapted GBS strains (Fig. 6). In the COG analysis, twenty-one functional categories were classified as differentially regulated. Translation, ribosomal structure and biogenesis (*n* = 40), cell wall/membrane/envelope biogenesis (*n* = 27), general functions (*n* = 27), carbohydrate metabolism and transport (*n* = 26) and energy production and conversion (*n* = 22) were the most common categories represented by the down-regulated proteins in fish-adapted GBS strains in comparison to the human isolate; amino acid metabolism (*n* = 27), transcription (*n* = 21) and replication, recombination and repair (*n* = 13) were the main functions identified as up-regulated (Fig. 7).

**Table 5:** List of differentially regulated proteins identified in all fish-adapted GBS strains compared to the NEM316 strain

Up/Down^a^	Accession^b^	Product^c^	SA16 fold-change^d^	SA20 fold-change^d^	SA53 fold-change^d^	SA81 fold-change^d^	SA95 fold-change^d^
Down	GBS_RS00535	50S ribosomal protein L6	−4.57	−3.45	− 4.69	− 4.37	− 1.78
Down	SaSA20_0140	Fe-S assembly protein NifU	−4.23	−2.33	−3.59	− 1.40	− 1.12
Down	GBS_RS01110	Acetate kinase	−2.95	−1.73	−2.03	− 1.42	− 1.15
Down	SaSA20_0551	Beta-lactamase	−5.34	−5.25	−4.93	− 5.62	−1.32
Down	SaSA20_0944	thrB Homoserine kinase	−4.67	−3.52	−2.85	− 3.46	− 4.69
Down	SaSA20_1545	Peptidase S66	−1.84	− 1.06	− 1.83	− 1.02	−1.16
Down	GBS_RS09900	Proline--tRNA ligase	−3.45	− 3.01	− 3.36	−2.68	− 2.93
Down	SaSA20_1738	DHH family phosphoesterase	−3.99	−2.65	−2.69	−1.84	− 2.15
Down	SaSA20_1009	Dihydroorotate dehydrogenase	−2.62	−2.13	−3.66	−1.22	−1.58
Down	SaSA53_1431	3-hydroxybutyryl-CoA dehydrogenase	−1.30	−2.33	−1.71	− 1.64	−1.21
Down	GBS_RS01730	Hypothetical protein	−4.30	−4.42	−3.84	− 4.19	−1.16
Up	GBS_RS07220	DNA (cytosine-5-)-methyltransferase	6.76	2.53	7.20	5.40	1.05
Up	GBS_RS07415	ABC transporter ATP-binding protein	2.06	3.34	1.40	3.30	2.38
Up	SaSA20_0474	mlcE	1.55	2.47	2.62	2.06	1.37
Up	SaSA20_0529	gyrB DNA gyrase subunit B	5.10	6.07	5.44	5.55	2.71
Up	SaSA20_1579	galE_2 UDP-glucose 4-epimerase	1.07	1.52	1.45	1.69	1.02
Up	SaSA20_0382	Reticulocyte binding protein	4.98	4.52	5.45	4.21	1.51
Up	SaSA20_0123	argH Argininosuccinate lyase	1.74	2.78	2.58	2.05	1.97
Up	SaSA95_0249	cglA Competence protein	6.16	7.67	6.80	6.31	5.25
Up	SaSA20_0173	Thioredoxin	3.14	3.43	3.52	3.71	1.03
Up	SaSA53_0400	Hypothetical protein	3.86	3.72	7.25	4.21	1.78
Up	SaSA20_0423	GNAT family acetyltransferase	3.36	5.04	5.44	5.31	1.94
Up	SaSA20_0437	asnA Aspartate--ammonia ligase	4.12	3.94	3.28	3.90	1.46
Up	SaSA20_0674	nagB Glucosamine-6-phosphate deaminase	4.61	5.11	6.03	3.37	2.54
Up	SaSA20_0995	LytR family transcriptional regulator	2.58	2.06	1.94	2.97	1.15
Up	SaSA20_1068	fni Isopentenyl-diphosphate delta-isomerase	4.28	4.82	4.21	5.04	1.77
Up	SaSA20_1080	Antibiotic ABC transporter ATP-binding protein	2.07	2.42	1.73	2.42	1.02
Up	SaSA20_1112	thiI tRNA sulfurtransferase	3.29	2.89	2.73	3.08	1.29
Up	SaSA20_1168	sigA RNA polymerase sigma factor	3.52	5.44	4.82	4.98	1.77
Up	SaSA20_1173	Amino acid ABC transporter substrate-binding protein	1.43	2.94	1.35	1.86	1.41
Up	SaSA20_1287	3-phosphoglycerate dehydrogenase	1.03	2.35	2.37	1.31	1.27
Up	SaSA20_1317	Non-canonical purine NTP pyrophosphatase	2.09	2.20	3.83	2.47	1.38
Up	SaSA53_1361	Non-canonical purine NTP pyrophosphatase	2.09	2.20	3.83	2.47	1.38
Up	SaSA20_1402	scrK Fructokinase	2.78	1.32	2.16	3.81	2.31
Up	SaSA20_1492	hypothetical protein	3.04	2.26	3.15	3.02	1.29
Up	SaSA20_1499	dltA	2.74	3.09	2.94	3.38	1.09
Up	SaSA20_1688	ABC transporter ATP-binding protein	8.13	7.87	7.46	7.26	3.66
Up	SaSA20_1692	Glyoxalase	2.32	3.16	2.77	3.06	1.43
Up	SaSA20_1762	arcB Ornithine carbamoyltransferase	2.85	2.82	2.71	3.01	1.09
Up	SaSA16_1675	Hypothetical protein	1.91	2.40	2.35	2.81	3.39

^a^Regulation of protein expression

^b^Locus tag of protein

^c^Protein product name

^d^Fold-change is the log2 of ratio between fish-adapted GBS strains and NEM316 proteins abundance

## Discussion

The fish-adapted GBS strains used in this study were isolated from fishes infected during outbreaks of meningoencephalitis among farm-raised Nile tilapia and Amazon catfish (*Leiarius marmoratus x Pseudoplatystoma fasciatum*) in Brazil on different farms between 2006 and 2010 (Table 1). As mentioned above, the GBS strains belong to serotype Ib, display different genetic profiles [10] and belong to two fish-adapted genotypic groups (the CC260 and NT lineages) that are closely related and that are commonly detected in fishes raised on Brazilian fish farms [9]. Although the selected strains display high similarity in genomic content (> 98%) and are considered very closely related, small variations between isolates with different STs have been verified [9]. Based on these considerations, comparison of the protein abundance among strains with different genotypes would demonstrate the pan-proteome of GBS fish-adapted strains. In addition, the NEM316 strain has been included in our study to allow the comparison with an isolate that not able to promote the streptococcal disease in fish hosts [32]. The NEM316 strain is a well-studied strain, with several works about your human-pathogenicity, including with transcriptomic and proteomic data [24, 29, 30].

The LC-UDMS^E^ label-free proteomic analysis conducted in our study resulted in the identification and quantification of 1070 proteins expressed by GBS strains. This is the largest number of proteins that have been identified for this bacterial species by proteomics, regardless of strain origin. A limited number (*n* = 65) of proteins was identified in previous studies of comparative proteome of GBS using two-dimensional electrophoresis (2-DE) combined with mass spectrometry [25, 48]. However, use of the LC-UDMS^E^ technique yielded better results for proteome analysis from whole bacterial lysates, as verified in our study. Of the total identified proteins, 1065 proteins represented the pan-proteome of the evaluated fish-adapted GBS strains; these proteins constituted approximately 60% of the predicted proteome of each strain.

The five fish-adapted GBS strains shared ~ 92% of their identified proteins, demonstrating that the expression of the core proteome is conserved among strains. Previous studies have also reported conservation of protein expression among isolates of the same bacterial species; in different studies, coverages ranging from 73.1 to 91.4% of the pan-proteome were reported [19, 27, 49, 50].

### Insights regarding the pan-proteome of fish-adapted GBS strains

#### Proteins involved in adaptation to an aquatic environment

Environmental factors such as the amount of dissolved oxygen, the pH, the osmotic strength, the temperature and the availability of nutrients can modify the expression of proteins in response to changes in these parameters. However, the proteins related to the survival of GBS in the aquatic environment are poorly characterized. It is known that GBS can be transmitted between fish indirectly through the water [4] and that increased expression of proteins involved in the transport of carbohydrates, amino acids and ions increases bacterial survival in this environment [14, 28].

Compounds such as glucose, mannitol, lactose, mannose and pyruvate are used as energy sources by *Streptococcus* species [51]; moreover, GBS has the capacity to utilize a broad range of carbon-containing molecules through the PTS system and ABC transporters [28]. Our study identified proteins involved in glycogen synthesis (PgmA), the glycolytic pathway (Eno, Pga, Pgk, and TpiA), the pentose phosphate pathway (AroD), 12 PTS system proteins involved in the transport of ascorbate, glucose, beta-glucoside, lactose, mannose, galactitol and fructose, and 2 sugar-specific ABC transporters (maltose ABC transporter substrate-binding protein and sugar ABC transporter ATP-binding protein). Taken together, the identification of these proteins demonstrated the broad catabolic capacity of GBS strains and corroborated the results obtained from genomic analysis performed by Glaser et al. [28]. Among the proteins involved in carbohydrate metabolism, TpiA and Pgk showed a high number of interactions in PPI analysis; they are also among the more abundant proteins in our proteomic data obtained from fish-adapted GBS strains.

In environments in which glucose or lactose availability is limited, pyruvate is thought to provide an alternative energy source for many bacterial species [52]. Proteins involved in pyruvate metabolism (pyruvate dehydrogenase, TPP-dependent acetoin dehydrogenase complex, branched-chain alpha-keto acid dehydrogenase and dihydrolipoyl dehydrogenase) were identified in the core proteome of fish-adapted GBS strains and formed part of an interactive network in PPI analysis. These four proteins make up the pyruvate dehydrogenase complex, which is responsible for the conversion of pyruvate into acetyl-CoA, an important precursor in fatty acid biosynthesis and a metabolic intermediate in acetate production [53]. The degradation of pyruvate by GBS strains generates products such as formate, acetate, acetoin, lactate and ethanol, which serve as carbon substrates for energy production [53–55]. Moreover, acetate kinase (AckA) and L-lactate dehydrogenase (Ldh_2), which were also identified in the core proteome, increase the formation of acetate and lactate, thereby generating more energy in the form of ATP, regenerating NAD from NADH in a reaction catalyzed by the NoxE protein, and permitting bacterial survival in aerobic and oxygen-depleted environments [49, 54].

Various amino acids have been considered essential for the growth of GBS strains under aerobic and anaerobic conditions [56]. The proteins involved in the metabolic pathways that produce glycine, serine, glutamine, aspartic acid, threonine, alanine and asparagine in the NEM316 strain have already been described through genomic analysis [28]. Proteins involved in these metabolic pathways were also identified in our study of fish-adapted GBS strains, as shown in Table 2. In addition, because GBS is an auxotrophic microorganism for the biosynthesis of some amino acids, it is necessary for it to produce the transport proteins and peptidases required to obtain these compounds in a nutrient-rich environment [28, 57]. Our proteomic data demonstrated that fish-adapted GBS strains express proteins related to the uptake of amino acids, including ABC transporters specific for amino acids and peptides (*n* = 25), peptidases (*n* = 29), and proteins involved in arginine, glutamic acid, cysteine and methionine metabolism, all of which are important for survival in aquatic and host environments.

Inorganic and metallic ions are important cofactors that contribute to the biological activities of many bacterial proteins [58]. These factors must be acquired from the environment [59]. However, in fish-adapted GBS strains, the genes involved in inorganic ion metabolism may be missing or inactivated, affecting ion exchange and reducing the bacterium’s ability to maintain homeostasis when exposed to changes in the external environment [14]. The results of our study are inconsistent with previous findings based on the comparative genomic analysis of seven GBS strains isolated from fish and frog hosts, since in our study the expression of 15 ABC transport proteins involved in the mobilization of iron, nickel, ferrichrome, manganese, magnesium, potassium, phosphate and heme, as well as proteins involved in zinc (zinc-binding protein) and copper (CutC) metabolism, was detected. These proteins may be important for the growth and survival of fish-adapted GBS strains in aquatic environments, which often contain a limited supply of essential metal ions. Some of these proteins, such as MscL and TrkA, also participate in bacterial cell osmoregulation. MscL activates the release of cytoplasmic solutes from mechanosensitive channels, decreasing the turgor pressure during changes in osmolarity [60], and TrkA participates in the uptake of potassium, an important inorganic ion required for the maintenance of constant bacterial internal pH and membrane potential [61, 62]. TrkA was down-regulated in fish-adapted GBS strains in comparison with NEM316, demonstrating that the strains isolated from fishes have a lower rate of potassium uptake.

Proteins involved in lipid metabolism in GBS have generally been poorly characterized; however, important proteins related to this functional category, including AccD, FabD, FabF, FabG, FabH, FabT and FabZ, were identified in our study. Among these proteins, FabT, a transcriptional regulator of the MarR family, has been shown to be associated with the control of membrane fatty acid composition and survival in low-pH environments in *Streptococcus pneumoniae* [63]. Through the generation of *fabT* mutant strains of *S. pneumoniae*, Lu and Rock [63] verified that up-regulation of the *fab* gene cluster by the inactivation of FabT leads to a deficiency in unsaturated fatty acids (UFA) and an increase in the proportion of 18-carbon fatty acids in the bacterial membrane, culminating in an acid-sensitive growth phenotype of the pathogen. Loss of UFA also resulted in sensitivity of *Streptococcus mutans* to acidic pH environments [64]. Therefore, this behavior seems to be intrinsic to the genus *Streptococcus*. Interestingly, our proteomic data showed that some Fab proteins (FabG and FabZ) were down-regulated in fish-adapted GBS strains in comparison to the human-adapted GBS strain, suggesting a high level of unsaturated fatty acids in the membranes of fish-adapted GBS strains; as a consequence, the membrane becomes more fluid, conferring greater bacterial resistance to acidic environments. It is known that fish-adapted GBS strains are able to grow at a wide range of pH (3 to 11) [65] and that the ability to survive low-pH conditions may be critical for these strains to persist in the aquatic environment. This is especially important considering that the water used in fish farms can sometimes be acidic (pH = 6.3 ± 0.3) [66] and that it may cause fish disease after oral entry and gastrointestinal colonization [67] by bacteria that are able to resist the low pH present in the stomach and the high pH present in the gut. In addition, considering that fishes are poikilothermic animals, the higher membrane fluidity of fish-adapted GBS strains may also improve bacterial survival in environments that feature constant thermal variation, such as the water in fish farms and in the host environment.

Because GBS outbreaks usually occur under conditions of high water temperature, water temperature has been considered a predisposing factor for the occurrence of GBS infection in fish [4]. The expression of genes and proteins related to thermal adaptation is a universal response observed in prokaryotes, and the transient induction of chaperonin, heat shock and cold shock proteins represents an important mechanism of protection and homeostasis through which such organisms cope with physiological and environmental stress at the cellular level [68]. One of the thermal adaptation proteins that showed differential expression in our study, ClpP, is involved in the regulation of GBS growth at high temperatures and in bacterial survival under various stress conditions [69]. Other thermal shock-associated proteins (DnaK, GroL, GroS, GrpE, Pnp, RNA helicase and cold-shock protein) prevent the inactivation of cellular proteins and assist in the degradation of non-repairable denatured proteins that accumulate during normal growth or under stress conditions [68]. These proteins were also visualized in our PPI analysis and showed a high number of interactions.

In summary, our pan-proteomic data on metabolic networks suggest that the identified proteins reflect an adaptive ability of fish-adapted GBS strains to response to an aquatic environment. The enhanced expression of these proteins broadens the catabolic capacity for energy generation, increases the diversity of transport system proteins and thereby permits the uptake of carbohydrates, amino acids and ions from water, and modulates the lipid composition of the bacterial membrane. Moreover, the identification of proteins involved in stress responses showed that fish-adapted GBS strains are capable of protecting themselves from a broad range of potential cellular damage that might otherwise be caused by environmental stressors.

### Proteins involved in host-pathogen interaction

The transition of GBS from the aquatic environment to fish tissues usually requires adaptive changes. One way for this pathogen to monitor and respond to its environment is through the use of proteins that work as part of a two-component signal transduction system [70]. Among the known proteins related to signal transduction, we identified CiaR, CovS/CovR and Stp1/Stk1. CiaR contributes to GBS survival in phagocytic and non-phagocytic cells and to virulence potential in a murine model experimentally infected with wild-type and mutant GBS strains [71]. Mutation of the *covS*/*covR* genes in a GBS strain reduced the hemolytic activity of the strain on blood agar and impaired bacterial viability in human serum [72], whereas mutations affecting Stp1/Stk1 impaired GBS growth, cell segregation and virulence in a neonatal rat sepsis model [73]. The expression of these proteins in fish-adapted GBS strains thus appears to improve bacterial survival and increase their dissemination in fish tissues due to increased bacterial survival in serum.

GBS causes septicemia and meningoencephalitis in fishes [4]; however, the pathogenesis of this disease is poorly understood. Although the genome of fish-adapted GBS strains shows the presence of several virulence genes that have already been reported and characterized in human GBS strains, little is known about the participation of these genes in the pathogenesis of the disease in fishes. The primary virulence factors described for GBS are adhesins, invasins and evasins. We identified some proteins for the first time in fish-adapted GBS strains; these included PavA (adhesion), GapN (adhesion), internalin (invasion), hemolysin A (invasion), several immune evasins (NeuABCD, CpsBCG, RmlABC, and serine protease) and penicillin-binding proteins (PbpX, Pbp1A and Pbp2A). These proteins have not yet been studied in terms of their biological functions in fish-adapted GBS strains, but several them have been very well characterized in human GBS strains [57]. The detection of these proteins in fish-adapted GBS strains suggests that their participation in pathogenesis is similar in aquatic hosts and mammalian hosts. An example of this is offered by the identification of the BibA and IagA proteins in our data. These two proteins are involved in GBS invasion and colonization of brain tissue in a murine model and in GBS survival in human blood [74, 75]. The identification of these proteins in fish-adapted GBS strains suggests their possible association with the clinical manifestations of disease under field conditions in which the diseased fish showed meningoencephalitis and septicemia. However, future research must to be conducted to validate this possibility.

Another identified protein in our study that contributes to bacterial adhesion is elongation factor Tu. This protein was shown to mediate the binding of bacteria to fibronectin, fibrinogen and mucin in studies of *Mycoplasma pneumoniae*, *Listeria monocytogenes* and *Lactobacillus johnsonii*, respectively [76–78]. Elongation factor Tu was previous identified in a proteomic study using fish-adapted GBS strains and shown to be highly expressed in a virulent strain [25]. Similarly, elongation factor Tu was the most abundant protein identified in our work.

Interestingly, some proteins involved in virulence were identified in the SA20-, SA53- and SA95-unique proteomes. These proteins might contribute to the pathogenesis of GBS in fishes. Abortive infection protein, an integrative and conjugative element involved in virulence and metal resistance in GBS [79], was identified in SA20*.* Virulence factor EsxA, which was identified in SA53, was shown to contribute to bacterial dissemination and colonization of *Streptococcus suis* in a mouse infection model [80] and to induce antibodies in humans infected with *Staphylococcus aureus* [81]. Another virulence protein identified in SA53 was gluconate 5-dehydrogenase, which catalyzes the reversible oxireduction of D-gluconate to 5-keto-D-gluconate [82]. D-gluconate is an important carbon source for prokaryotes and is involved in the colonization, survival and virulence of *E. coli* in streptomycin-treated mice [83] and in cell division in *S. suis* [84]. In the SA95-unique proteome, glycosyl transferase was identified. This protein belongs to a class of enzymes that are responsible for the formation of structural molecules such as glycoproteins, glycolipids, oligosaccharides and of the cell wall and that also act in immune recognition, bacterial evasion, intercellular signaling and biofilm formation [85].

Other proteins identified in our pan-proteome data, such as those involved in nucleotide metabolism and oxidative stress, may also contribute to the pathogenicity of GBS in fishes. A previous study demonstrated that purine and pyrimidine metabolism is essential for the survival and growth of *Escherichia coli*, *Salmonella enterica* and *Bacillus anthracis* in human serum [86]. In GBS, on the other hand, genes involved in purine and pyrimidine metabolism showed significant modification of transcription in response to incubation with human blood, revealing a dynamic metabolic adaptation of this bacterium [29]. In our PPI analysis, numerous interactions between proteins related to nucleotide metabolism were detected. Therefore, after fish infection, the expression of proteins involved in nucleotide metabolism may be associated with GBS serum resistance in the fish host, as previously demonstrated by Wang et al. [87].

During the infection process, bacteria encounter reactive oxygen species (ROS) generated by neutrophils and macrophages of the host as a defense mechanism; these ROS directly damage proteins, nucleic acids and other cellular components [53, 88]. We identified the expression of proteins involved in ROS detoxification, including SodA, SufB, SufC, SufD, TrxB, thioredoxin and NoxE, that have been previously characterized in GBS strains [28, 89]. Among these proteins, superoxide dismutase (SodA) is also involved in virulence, contributing to the pathogenicity of GBS by allowing bacterial survival in macrophages and maintaining a high bacterial load in the blood of experimentally infected mice [90].

### Putative vaccine targets

Due to the high similarity of the genomic content of the GBS strains used in this work, a predicted vaccine candidate for all strains could reasonably be expected to confer protection against the disease regardless of the circulating genotype in a fish farm.

Eleven of the 38 predicted antigenic proteins were also detected in a previous study of conserved antigenic proteins in GBS strains isolated from human (*n* = 10), bovine (*n* = 1) and fish (*n* = 4) hosts [91] as being shared only by fish-adapted GBS strains. Among these proteins, the immunogenicity and efficacy of a recombinant vaccine against GBS prepared against the cell wall surface anchor protein has already been evaluated in tilapia and turbot that were vaccinated and experimentally infected [92]. Although the evaluation was performed using high doses (10^8^ CFU fish^− 1^) of a fish-adapted GBS strain, the vaccine provided relatively high percentage survival (RPS) of 72.5 and 72.7% for tilapia and turbot, respectively [92].

An important putative vaccine target in our study was Sip. This protein is highly conserved among all GBS strains regardless of serotype [93] and was one of the most abundant proteins in our pan-proteome data (Fig. 1). Moreover, Sip has been used in the preparation of vaccines against GBS in tilapia, resulting in an RPS of 41.6 to 95.8% using DNA or adjuvanted vaccines and conferring high protection in vaccinated fish [93, 94].

Other predicted proteins with unknown functions (hypothetical proteins) and proteins that have not yet been tested as vaccine targets may be used for vaccine development in further studies aimed at evaluating their potential for the protection of fishes against GBS infection and to determine whether they confer immunity to strains belonging to different clonal complexes.

### Global differential expression of proteins

To explore changes in protein abundance linked to host adaptation, we performed a comparative proteome analysis of human and fish-adapted GBS strains. This type of comparison was previously performed using a microarray approach; the results obtained using that approach showed that there is a closer genetic relationship between the GBS CF01173 strain isolated from fish (ST-7) and the A909 strain isolated from human (ST-7) and indicated genetic divergence of the strains 2–22 (ST-261) and SS1219 (ST-260) from strain ST-7 at the transcriptional level [14]. However, the proteomic approach is more robust than the microarray technique for evaluating the expression of the functional genome because it measures the expression of proteins that are directly involved in enzymatic catalysis, molecular signaling, and physical interactions [95].

One protein present in the NEM316-unique proteome was associated with specialization of the bacterium to the human host. This transcriptional regulator (GBS_RS10725) is present only in the genome of the NEM316 strain, having been deleted during reductive evolution of the ST260–261 strains [14]. This protein is a positive regulator of resistance to cadmium in some GBS strains [96]. On the other hand, the proteins that were exclusively identified in fish-adapted GBS strains are related mainly to virulence factors that have already been discussed in this work and that together may increase the possibility of onset of disease in fish. However, our qualitative proteomic analysis of human- and fish-adapted GBS strains did not indicate the basis for the host specificity of the strains, as highly similar protein content was observed in all of the examined strains. A possible reason for the exclusive detection of these virulence proteins in fish-adapted GBS might be the temperature-independent regulation of several genes from this category on fish-adapted strains, as described in a recent study of our group [97]. Conversely, the strain NEM316 showed a distinct behavior of high expression of virulence genes at 40 °C when compared with low temperature conditions (i.e., 30 °C) [30].

Although the protein content of human and fish-adapted GBS strains was similar, there was differential expression at the proteome level. An association between the level of expression of specific proteins and the genotype of fish-adapted strains was observed. In all fish-adapted GBS strains, 40, 27, 26 and 22 proteins involved in translation, ribosomal structure and biogenesis, cell wall/membrane/envelope biogenesis, carbohydrate transport and metabolism and energy production and conversion, respectively, were expressed at lower levels than in the NEM316 strain. These results reveal a reduced catabolic capacity of fish-pathogenic *S. agalactiae* in comparison with the human GBS strain. Previous studies using genomic approaches have suggested that the reduction of catabolic capacity in fish-adapted strains could be linked to adaptation of the bacterium to aquatic hosts [14, 98].

Among the proteins identified as DEPs in fish- and human-adapted GBS strains (Table 5), reticulocyte binding protein showed increased expression (its log_2_ ratio increased from 1.51 to 5.45) compared with the NEM316 strain. This protein is a serine protease that is homologous to C5a peptidase (ScpB), which facilitates host immune evasion through cleavage and inactivation of complement component C5a and promotes adhesion to host cells [57]. In addition, proteins involved in multidrug resistance, such as DltA, antibiotic ABC transporter ATP-binding protein and GNAT family acetyltransferase, were expressed at log_2_ ratios 1.02–5.44-fold higher in fish-adapted GBS strains than in the NEM316 strain. The up-regulation of these proteins might modulate host cellular processes, especially the complement cascade and the IFN pathway, both of which are considered effective defenses against bacterial pathogens [99] and are known to contribute to the adhesion, dissemination, and persistence of GBS in various fish tissues. On the other hand, 11 proteins were down-regulated in fish-adapted GBS strains compared to the NEM316 strain; beta-lactamase (log_2_ ratio of − 1.32 to − 5.62), ThrB (− 2.85 to − 4.69) and PavA (− 1.22 to − 3.66) showed higher expression in the latter strain. Despite the identification of virulence proteins that showed differential regulation in the human GBS strain, our results are not consistent with the results of in vivo trials previously performed by our group in which it was shown that fish-adapted GBS strains cause mortality in Nile tilapia whereas the NEM316 strain causes a transient infection in which fish do not manifest clinical signs of disease or mortality [32]. However, some of the differentially expressed proteins, such as PavA, modulate the activity of important virulence factors in *Streptococcus pneumoniae* that are associated with adherence and survival in experimentally infected mice; thus, even a wild-type strain that expresses the *pavA* gene may cause higher mortality than that caused by the isogenic mutant [100]. Therefore, the proteins that are up-regulated in the NEM316 strain might be active in GBS virulence only in the mammalian host and may not contribute to disease in aquatic animals.

## Conclusions

The current study is the first to evaluate the whole proteome of GBS strains by LC-UDMS^E^; it is also the first study to compare the proteome of this pathogen in different, closely related genotypes. Our results demonstrated high similarity of the expressed proteins and showed that the core proteome of fish-adapted GBS strains is conserved. Our comparison of protein expression among isolates with different genotypes belonging to fish-associated clonal complexes provided information about the metabolism, the survival strategy, the adaptation and the pathogenicity of fish-pathogenic GBS strains. The high degree of conservation among strains with different STs suggests that monovalent vaccines may be effective against different genetic variants within clonal complexes.

## Additional files

Additional file 1: **Figure S1.** Quality control of the proteins identified by LC-MS. A: normal distribution of 10 ppm error of the total identified peptides. B: peptide detection type. PepFrag1 and PepFrag2 correspond to the peptide matches obtained by comparison to the database by Progenesis QIP, VarMod corresponds to variable modifications, InSource corresponds to fragmentation that occurred at the ionization source, and MissedCleavage corresponds to missed trypsin cleavage. C: drift time for ions of 1+ (blue), 2+ (green), 3+ (red), 4+ (yellow) and 5+ (purple) charge states. D: repeat rate indicating the number of times that an identified protein appears in the replicates: 3 of 3 (blue) and 2 of 3 (red). E: principal component analysis, biological replicates (*n* = 3) of GBS strains; NEM316 (red), SA16 (blue), SA20 (yellow), SA53 (gray), SA81 (green) and SA95 (black). (PDF 364 kb)

Additional file 2: **Table S1.** Complete list of proteins identified by LC-UDMS^E^. (XLSX 1045 kb)

Additional file 3: **Table S2.** Enrichment analysis using KEGG pathways in the STRING web tool. (DOCX 14 kb)

Additional file 4: **Table S3.** Exclusive proteins identified in human and fish-adapted GBS strains. (DOCX 14 kb)

Additional file 5: **Table S4.** Number of proteins differentially expressed between fish-adapted GBS strains and the NEM316 strain. (DOCX 15 kb)

Additional file 6: **Figure S2.** Venn diagram showing the number of proteins up- and down-regulated in fish-adapted GBS strains in comparison to the NEM316 strain. (PDF 149 kb)

Additional file 7: **Table S5.** Complete list of proteins that were up-regulated and down-regulated in fish-adapted and human GBS strains. (XLSX 91 kb)

## Abbreviations

2-DE: Two-dimensional electrophoresis
ATCC: American type culture collection
BHI: Brain heart infusion
CC: Clonal complex
CDS: Protein code sequences
CHAPS: 3-[(3-Cholamidopropyl)dimethylammonio]-1-propanesulfonate hydrate
COG: Cluster of orthologous genes
DEP: Differentially expressed proteins
DIA: Data-independent acquisition
DTT: Dithiothreitol
FDR: False discovery rate
FWHM: Full width at half-maximum
GBS: Group B *Streptococcus* or *Streptococcus agalactiae*
Glu-Fib: [Glu^1^]-fibrinopeptide B
KEGG: Kyoto encyclopedia of genes and genomes
MLST: Multilocus sequence typing
nanoESI-HDMS^E^: Nano electrospray high-definition mass spectrometry
nanoLC-UDMS^E^: Nano-liquid chromatography-ultra definition mass spectrometry
nanoUPLC: Nano ultra-performance liquid chromatography
NT: Non-typeable
*oa*-TOF: Orthogonal acceleration time-of-flight
OD: Optical density
PCA: Principal component analysis
PepFrag1: First pass of peptides identification
PepFrag2: Second pass of peptides identification
PPC: Predicted protein clustering
PPI: Protein-protein interaction
ppm: Parts per million
PSE: Potentially surface-exposed
PTS: Phosphoenolpyruvate-dependent phosphotransferase
ROS: Reactive oxygen species
RP: Reverse-phase
RPS: Relative percentage survival
ST: Sequence type
Tris HCl: Tris hydrochloride
UFA: Unsaturated fatty acids
VarMod: Variable modifications
