# Supplementary material for: Delineation of the pan-proteome of fish-pathogenic Streptococcus agalactiae strains using a label-free shotgun approach
Source: BMC Genomics. 2019 Jan 7;20:11. doi: 10.1186/s12864-018-5423-1 (PMC6323687; doi:10.1186/s12864-018-5423-1)
Supplement: Supplementary file 1 — Figure S1. Quality control of the proteins identified by LC-MS. A: normal distribution of 10 ppm error of the total identified peptides. B: peptide detection type. PepFrag1 and PepFrag2 correspond to the peptide matches obtained by comparison to the database by Progenesis QIP, VarMod corresponds to variable modifications, InSource corresponds to fragmentation that occurred at the ionization source, and MissedCleavage corresponds to missed trypsin cleavage. C: drift time for ions of 1+ (blue), 2+ (green), 3+ (red), 4+ (yellow) and 5+ (purple) charge states. D: repeat rate indicating the number of times that an identified protein appears in the replicates: 3 of 3 (blue) and 2 of 3 (red). E: principal component analysis, biological replicates (n = 3) of GBS strains; NEM316 (red), SA16 (blue), SA20 (yellow), SA53 (gray), SA81 (green) and SA95 (black). (PDF 364 kb) [file 12864_2018_5423_MOESM1_ESM.pdf]

Additional file 1: Figure S1. Quality control of the proteins identified by LC-MS. A: normal distribution of 10 ppm error of the total identified peptides. B: peptide detection type. PepFrag1 and PepFrag2 correspond to the peptide matches obtained by comparison to the database by Progenesis QIP, VarMod corresponds to variable modifications, InSource corresponds to fragmentation that occurred at the ionization source, and MissedCleavage corresponds to missed trypsin cleavage. C: drift time for ions of 1+ (blue), 2+ (green), 3+ (red), 4+ (yellow) and 5+ (purple) charge states. D: repeat rate indicating the number of times that an identified protein appears in the replicates: 3 of 3 (blue) and 2 of 3 (red). E: principal component analysis, biological replicates (n = 3) of GBS strains; NEM316 (red), SA16 (blue), SA20 (yellow), SA53 (gray), SA81 (green) and SA95 (black).

A

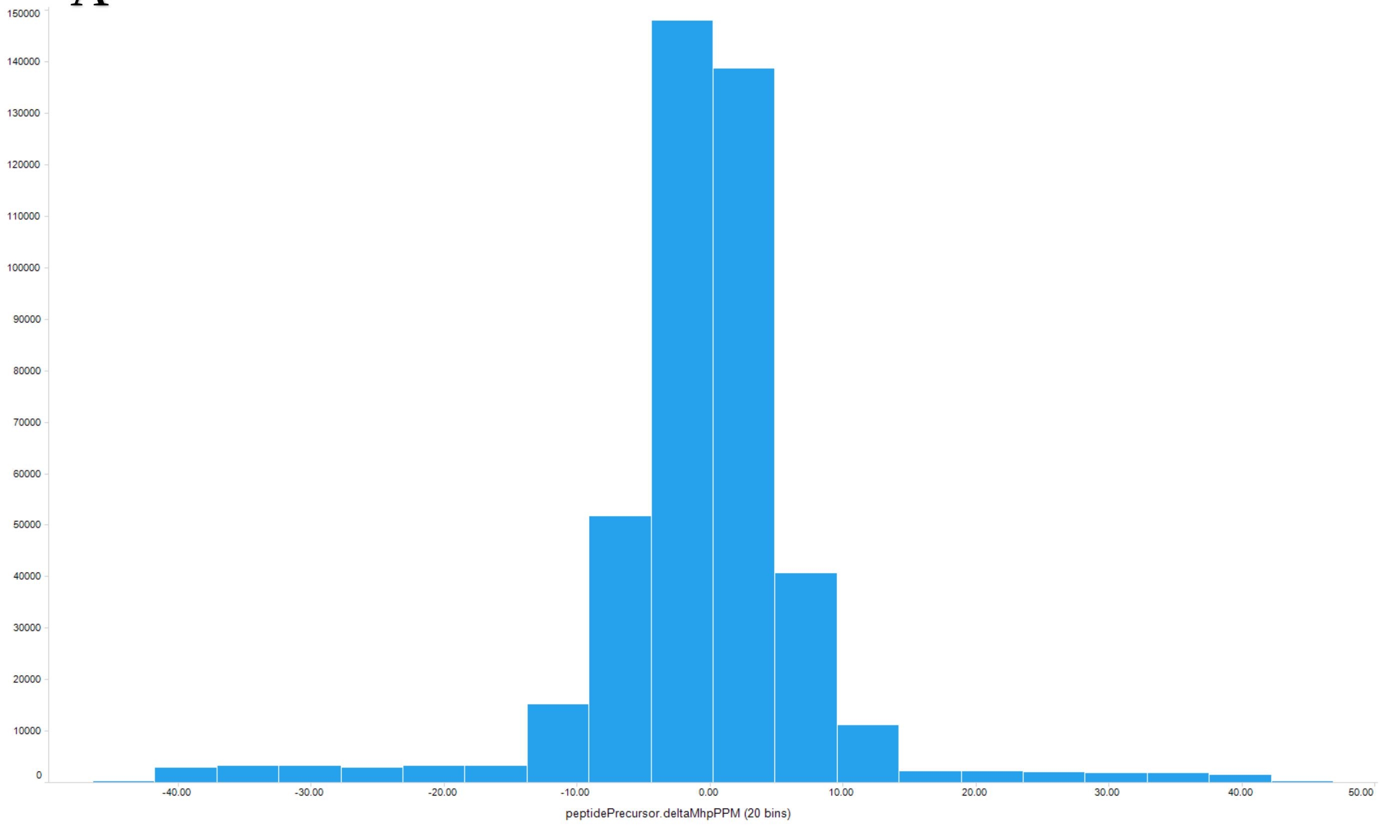

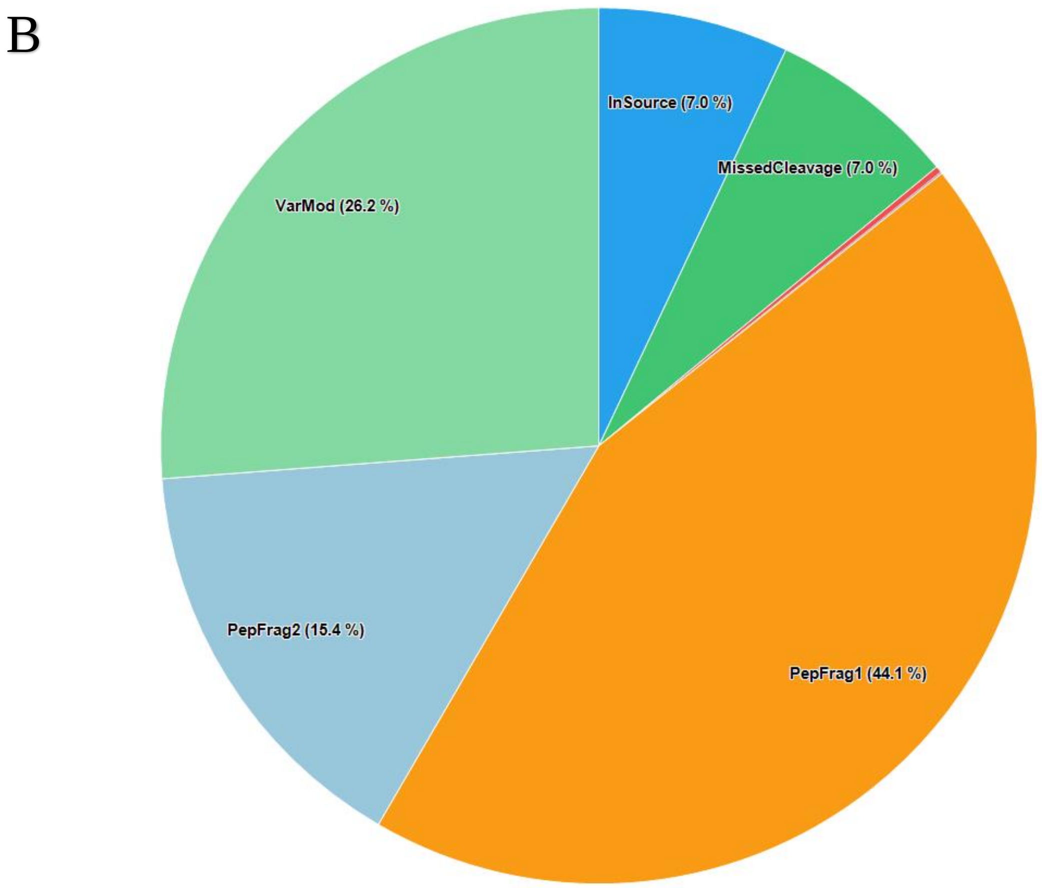
B
InSource (7.0 %)
MissedCleavage (7.0 %)
VarMod (26.2 %)
PepFrag2 (15.4 %)
PepFrag1 (44.1 %)

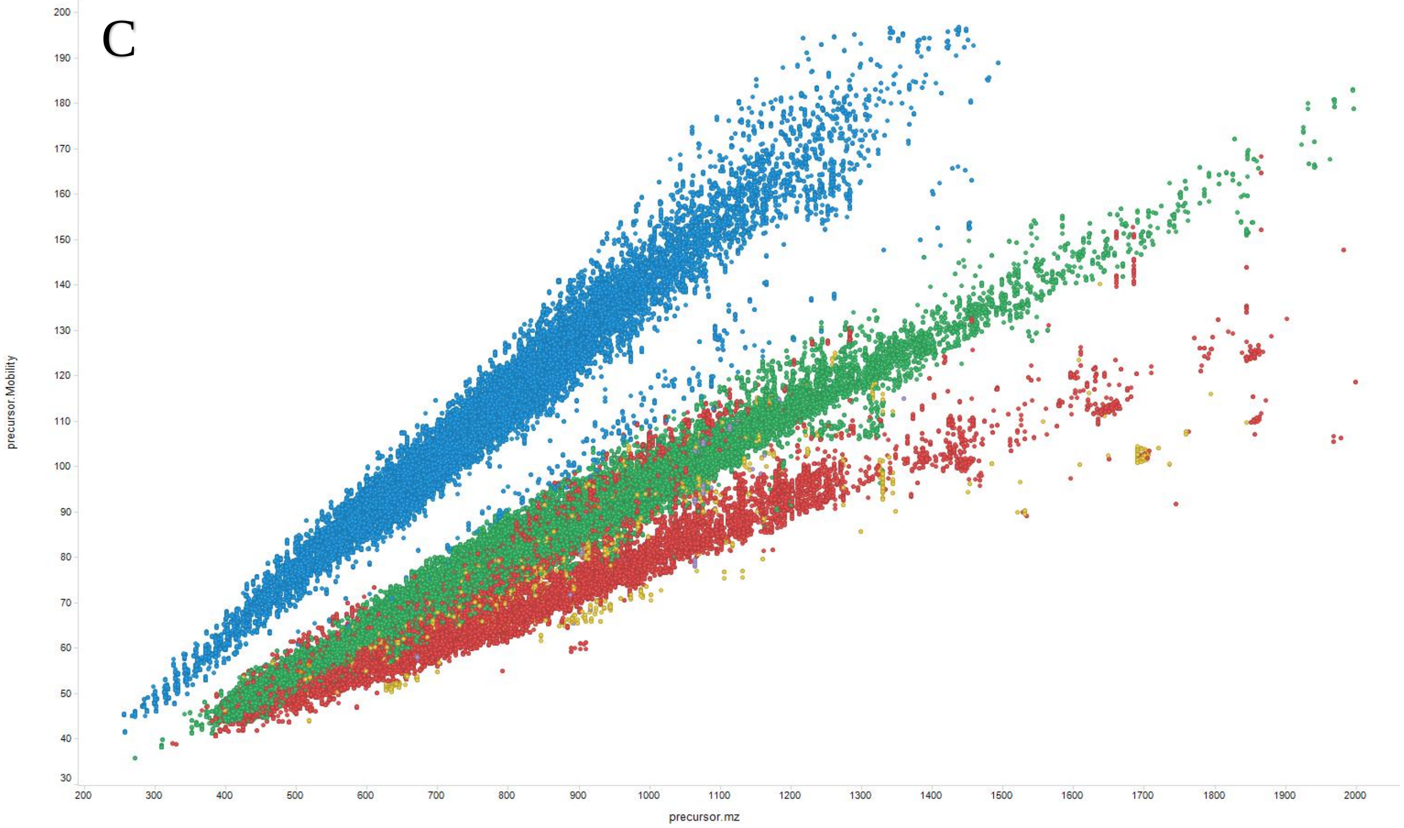
C
precursor.Mobility
precursor.mz
200
190
180
170
160
150
140
130
120
110
100
90
80
70
60
50
40
30
200 300 400 500 600 700 800 900 1000 1100 1200 1300 1400 1500 1600 1700 1800 1900 2000

D

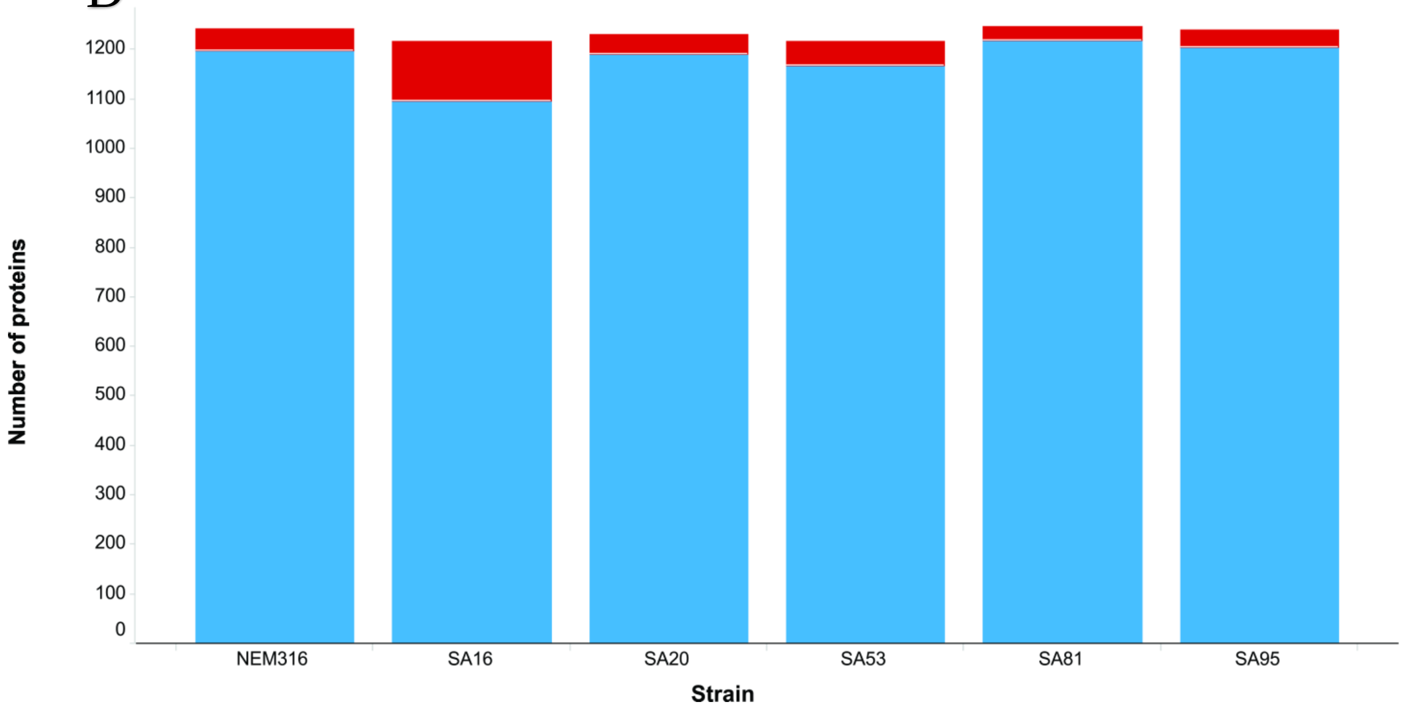

Number of proteins
1200
1100
1000
900
800
700
600
500
400
300
200
100
0
NEM316
SA16
SA20
SA53
SA81
SA95
Strain

E

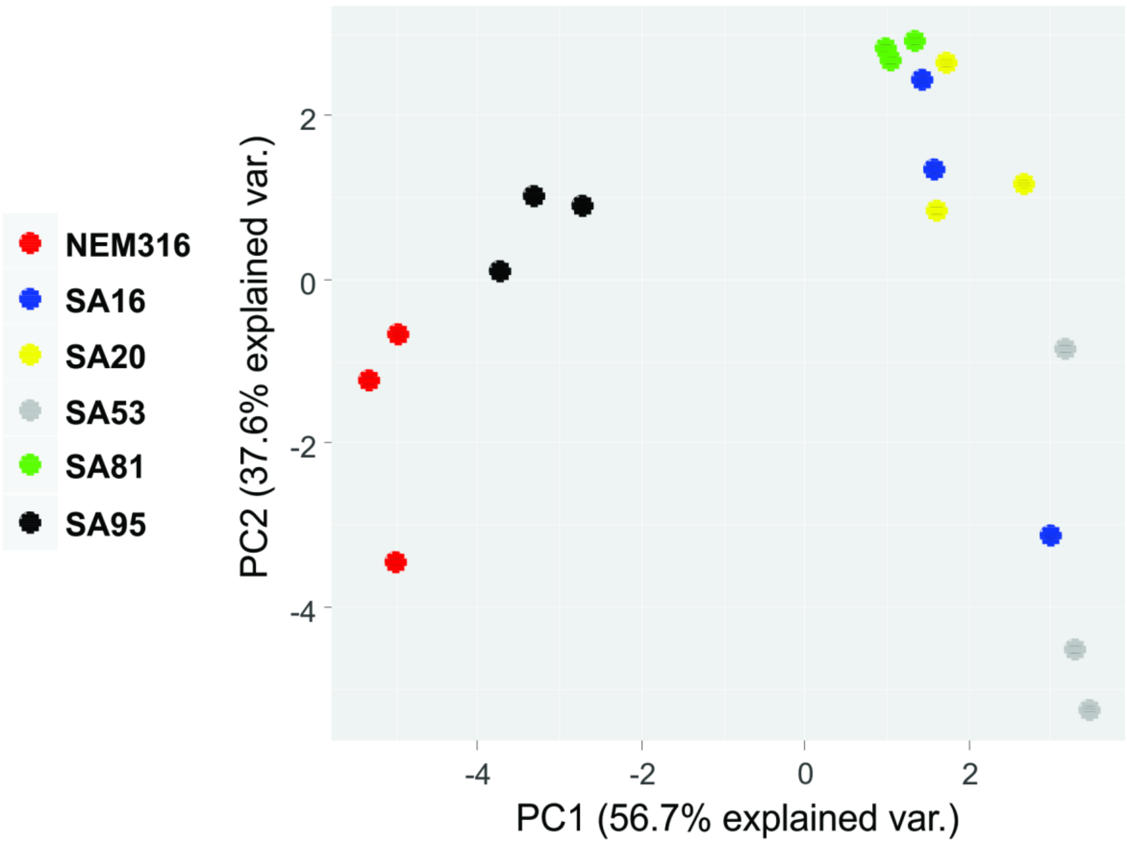

NEM316
SA16
SA20
SA53
SA81
SA95
PC2 (37.6% explained var.)
2
0
-2
-4
-4
-2
0
2
PC1 (56.7% explained var.)
