# Supplementary material for: Delineation of the pan-proteome of fish-pathogenic Streptococcus agalactiae strains using a label-free shotgun approach
Source: BMC Genomics. 2019 Jan 7;20:11. doi: 10.1186/s12864-018-5423-1 (PMC6323687; doi:10.1186/s12864-018-5423-1)
Supplement: Supplementary file 3 — Table S2. Enrichment analysis using KEGG pathways in the STRING web tool. (DOCX 14 kb) [file 12864_2018_5423_MOESM3_ESM.docx]

**Additional file 3: Table S2.** Enrichment analysis using KEGG pathways in the STRING web tool.

| Pathway description | Nº of proteins | FDR |
| --- | --- | --- |
| Metabolic pathways | 232 | 6.88e-36 |
| Biosynthesis of secondary metabolites | 104 | 2.37e-23 |
| Ribosome | 51 | 3.82e-16 |
| Purine metabolism | 48 | 1.03e-10 |
| Carbon metabolism | 43 | 8.92e-10 |
| Biosynthesis of amino acids | 48 | 8.92e-10 |
| Aminoacyl-tRNA biosynthesis | 25 | 1.2e-09 |
| Microbial metabolism in diverse environments | 61 | 1.47e-09 |
| Amino sugar and nucleotide sugar metabolism | 27 | 2.7e-06 |
| Glycolysis / Gluconeogenesis | 23 | 3.4e-06 |
| Pyrimidine metabolism | 36 | 5.81e-06 |
| Glycine, serine and threonine metabolism | 16 | 3.2e-05 |
| Methane metabolism | 13 | 0.000392 |
| Peptidoglycan biosynthesis | 18 | 0.000423 |
| Pyruvate metabolism | 18 | 0.000423 |
| Arginine and proline metabolism | 12 | 0.000748 |
| RNA degradation | 8 | 0.00341 |
| Starch and sucrose metabolism | 14 | 0.00585 |
| Mismatch repair | 14 | 0.00585 |
| Streptomycin biosynthesis | 7 | 0.00727 |
| Pentose phosphate pathway | 16 | 0.00792 |
| One carbon pool by folate | 8 | 0.0153 |
| Riboflavin metabolism | 6 | 0.0158 |
| Homologous recombination | 14 | 0.0236 |
| Nicotinate and nicotinamide metabolism | 7 | 0.0303 |
| Cysteine and methionine metabolism | 12 | 0.0346 |
| RNA polymerase | 5 | 0.0346 |
| Bacterial secretion system | 9 | 0.0484 |
