# Supplementary material for: Delineation of the pan-proteome of fish-pathogenic Streptococcus agalactiae strains using a label-free shotgun approach
Source: BMC Genomics. 2019 Jan 7;20:11. doi: 10.1186/s12864-018-5423-1 (PMC6323687; doi:10.1186/s12864-018-5423-1)
Supplement: Supplementary file 4 — Table S3. Exclusive proteins identified in human and fish-adapted GBS strains. (DOCX 14 kb) [file 12864_2018_5423_MOESM4_ESM.docx]

**Additional file 4: Table S3.** Exclusive proteins identified in human and fish-adapted GBS strains.

| Accession | Cluster | Description | Host Exclusive in |
| --- | --- | --- | --- |
| SaSA20_0369 | Cluster0329 | 5-formyltetrahydrofolate cyclo-ligase | Fish |
| SaSA53_0506 | Cluster0420 | Hypothetical protein | Fish |
| GBS_RS05625 | Cluster0685 | Ribonuclease HII | Fish |
| SaSA20_0983 | Cluster0788 | N-acetyl neuramic acid synthetase NeuB | Fish |
| SaSA20_0989 | Cluster0793 | Polysaccharide biosynthesis protein CpsG | Fish |
| SaSA20_1480 | Cluster1201 | 3'-5' exoribonuclease | Fish |
| SaSA20_1574 | Cluster1277 | Bacteriocin transport accessory protein | Fish |
| GBS_RS09930 | Cluster1278 | Malate dehydrogenase | Fish |
| SaSA20_1598 | Cluster1294 | Flavoprotein | Fish |
| SaSA20_1606 | Cluster1302 | PTS mannose transporter subunit IIB | Fish |
| SaSA20_1629 | Cluster1316 | Hypothetical protein | Fish |
| SaSA16_0843 | Cluster1475 | PhoB family transcriptional regulator | Fish |
| SaSA20_0863 | Cluster1562 | Hypothetical protein | Fish |
| SaSA20_1453 | Cluster1580 | Phenazine biosynthesis protein PhzF | Fish |
| SaSA53_0595 | Cluster1713 | Gluconate 5-dehydrogenase | Fish |
| SaSA16_1239 | Cluster1728 | Accessory Sec system protein Asp1 | Fish |
| GBS_RS07210 | Cluster1749 | Hypothetical protein | Fish |
| GBS_RS06055 | Cluster1759 | ATPase AAA | Fish |
| SaSA20_1265 | Cluster1780 | Bleomycin resistance protein | Fish |
| GBS_RS01380 | Cluster1823 | Hypothetical protein | Fish |
| GBS_RS03805 | Cluster1906 | Beta-hexosamidase | Fish |
| SaSA20_0640 | Cluster0512 | Penicillin-binding protein 2B | Fish |
| SaSA53_1568 | Cluster1236 | Membrane protein | Fish |
| GBS_RS05750 | Cluster1939 | Type VII secretion protein EsaA | Fish |
| SaSA20_0091 | Cluster0101 | D-alanyl-D-alanine carboxypeptidase | Fish |
| SaSA16_1707 | Cluster1339 | cAMP factor | Fish |
| GBS_RS00775 | Cluster0115 | CTP synthetase | Human |
| SaSA20_0108 | Cluster0118 | DNA repair protein radA | Human |
| SaSA20_0127 | Cluster0135 | Hypothetical protein | Human |
| GBS_RS07805 | Cluster0929 | Nucleotide sugar dehydratase | Human |
| GBS_RS10725 | Cluster2091 | Transcriptional regulator | Human |
