# Supplementary material for: Delineation of the pan-proteome of fish-pathogenic Streptococcus agalactiae strains using a label-free shotgun approach
Source: BMC Genomics. 2019 Jan 7;20:11. doi: 10.1186/s12864-018-5423-1 (PMC6323687; doi:10.1186/s12864-018-5423-1)
Supplement: Supplementary file 5 — Table S4. Number of proteins differentially expressed between fish-adapted GBS strains and the NEM316 strain. (DOCX 15 kb) [file 12864_2018_5423_MOESM5_ESM.docx]

**Additional file 5: Table S4.** Number of proteins differentially expressed between fish-adapted GBS strains and the NEM316 strain.

| Strain | Up-regulated | Down-regulated | Total |
| --- | --- | --- | --- |
| SA16  SA20  SA53  SA81  SA95 | 127  134  159  119  58 | 203  186  199  153  35 | 330  320  358  272  93 |
