# Supplementary material for: Delineation of the pan-proteome of fish-pathogenic Streptococcus agalactiae strains using a label-free shotgun approach
Source: BMC Genomics. 2019 Jan 7;20:11. doi: 10.1186/s12864-018-5423-1 (PMC6323687; doi:10.1186/s12864-018-5423-1)

Additional file 6: Figure S2. Venn diagram showing the number of proteins up- and down-regulated in fish-adapted GBS strains in comparison to the NEM316 strain.

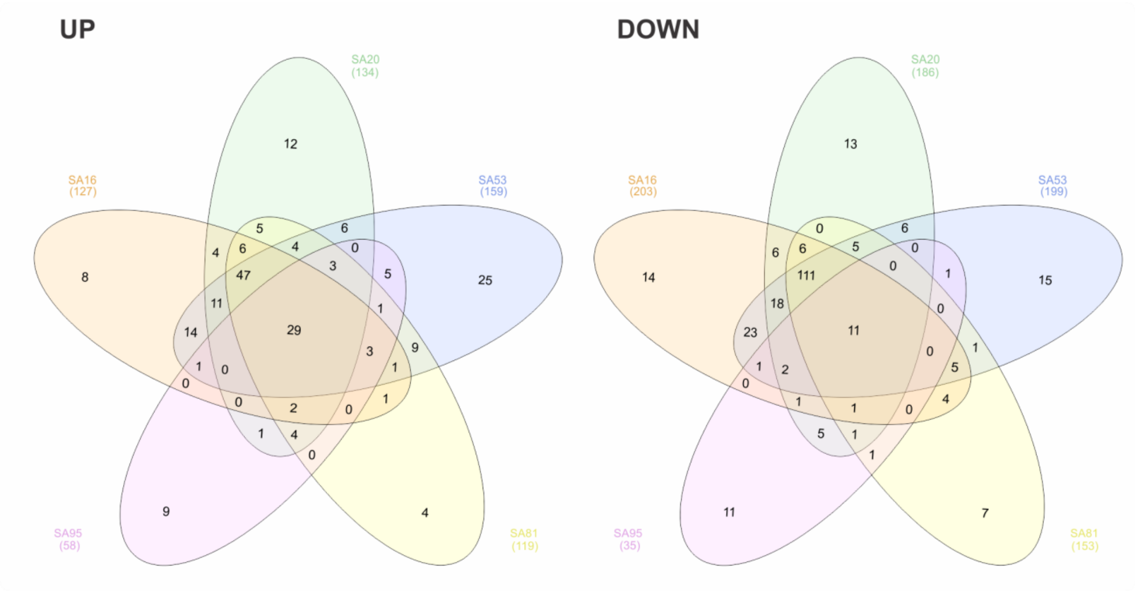

Supplement: Supplementary file 6 — Figure S2. Venn diagram showing the number of proteins up- and down-regulated in fish-adapted GBS strains in comparison to the NEM316 strain. (PDF 149 kb) [file 12864_2018_5423_MOESM6_ESM.pdf]
